# Electrocatalytic Nitrate Reduction to Ammonia Synthesis: Reaction Mechanisms, Catalytic Materials, and Future Perspectives

**DOI:** 10.3390/ma19163417

**Published:** 2026-08-12

**Authors:** Xuepeng Ni, Na Wei, Shanshan Guo, Zhenjiang Zhang, Yongtao Wang, Caixia Ren, Zhe Cui

**Affiliations:** 1 School of Energy and Constructional Engineering, Shandong Huayu University of Technology, Dezhou 253034, China; 2 Shandong Key Laboratory of Intelligent Manufacturing Technology for Advanced Power Equipment, Weifang University, Weifang 261061, China; 3 Christopher Ingold Laboratory, Department of Chemistry, University College London, London WC1H 0AJ, UK

**Keywords:** electrocatalysis, nitrate reduction, ammonia synthesis, electrocatalysts

## Abstract

The large-scale production and utilization of nitrogen-containing compounds have greatly promoted the development of modern agriculture and the chemical industry, but have also resulted in increasingly severe nitrate contamination and an imbalance of the nitrogen cycle. The efficient conversion of nitrate into value-added ammonia not only contributes to pollutant remediation but also provides a promising route for green ammonia synthesis. Owing to its mild reaction conditions, potentially lower environmental impact, and compatibility with renewable electricity, electrocatalytic nitrate reduction to ammonia has attracted considerable attention in recent years. This process involves a multielectron transfer process involving numerous intermediate transformations, and its catalytic performance largely depends on the adsorption and conversion of key intermediates on the catalyst surface, as well as the suppression of the competing hydrogen evolution reaction. This review systematically summarizes recent advances in electrocatalytic nitrate reduction to ammonia, with emphasis on the reaction mechanisms and major reaction pathways, as well as the design strategies, structure–activity relationships, and performance enhancement mechanisms of metal-based, carbon-based, and composite catalysts. In addition, the main challenges in this field, including product selectivity, mass transport, in-situ mechanistic characterization, and long-term stability, are discussed. Finally, the construction of highly efficient catalytic systems and key directions for future research are outlined, with particular emphasis on nitrate valorization and sustainable ammonia synthesis.

## 1. Introduction

Ammonia (NH_3_) is one of the most widely produced chemicals globally, being used primarily in fertilizer production (approximately 80%) and the production of nitrogen-containing chemicals such as nitric acid, other nitrogen-containing chemicals, urea, and amino acids [1,2]. In addition, due to its high energy density, high hydrogen content, and ease of liquefaction, storage, and transport, NH_3_ is regarded as a highly promising renewable energy carrier [3,4]. Therefore, developing an economical and efficient NH_3_ synthesis technology is of great significance for the sustainable development of human society. Currently, industrial NH_3_ synthesis relies mainly on the Haber–Bosch (H–B) process. This process operates under harsh conditions of high temperature (400–600 °C) and high pressure (200–350 atm), consuming approximately 1.4% of global annual energy and producing about 450 million tons of CO_2_, accounting for 1.2% of global annual CO_2_ emissions [5,6]. To address the high energy consumption and carbon emissions of the H–B process, the electrochemical nitrogen reduction reaction (eNRR) has been extensively studied as a potential alternative. However, the high dissociation energy of the triple bond in N_2_ (945 kJ mol^−1^) and its low solubility in aqueous solutions make the effective cleavage of N_2_ extremely difficult [7,8,9,10]. Moreover, the efficiency of eNRR for NH_3_ synthesis is low, and various byproducts are easily generated, posing significant challenges for accurate quantitative analysis of NH_3_ [11,12].

In recent years, researchers have proposed an alternative strategy: first oxidizing N_2_ to nitrogen oxides (NO_x_) or nitrates (NO_3_^−^), then converting them to NH_3_ via reduction reactions. Compared with N_2_, the N–O bond dissociation energy in NO_3_^−^ is significantly lower (204 kJ mol^−1^), and the conversion can be achieved under ambient conditions, providing an ideal pathway to replace the energy-intensive and high-carbon-emission H–B process [13,14]. Nitrate is widely present in domestic sewage and industrial wastewater. Its reduction technologies mainly include biological methods, chemical reduction, electrocoagulation, electrodialysis, and electrocatalytic reduction. However, biological methods suffer from slow reaction rates and sensitivity to environmental conditions [15,16]; chemical reduction requires H_2_ as a reducing agent, posing safety risks and leading to excessive accumulation of NH_4_^+^ [17,18,19]; electrocoagulation has high operating costs, generates metal hydroxide sludge, and is prone to the formation of passivation films on electrode surfaces [20,21]; electrodialysis is difficult to apply to low-concentration solutions due to high resistance, high energy consumption, and polarization phenomena [22].

In contrast, the electrocatalytic nitrate reduction reaction (eNO_3_^−^RR) has attracted intensive research interest in recent years due to its high reaction rate, mild reaction conditions, and simple reaction system. This review systematically summarizes the recent advances in eNO_3_^−^RR for NH_3_ synthesis, including: (1) the reaction mechanism of electrocatalytic nitrate reduction to ammonia; (2) the design and performance of various catalysts, such as monometallic, bimetallic, metal oxides, phosphides, sulfides, nitrides, and non-metallic catalysts; (3) the remaining challenges and future directions in this field. By summarizing existing research findings, this review aims to provide mechanistic insight and catalyst-design guidance for the further development of eNO_3_^−^RR technology.

## 2. Discussion on the Reaction Mechanism of Electrocatalytic Nitrate Reduction to Ammonia

The eNO_3_^−^RR to NH_3_ is a process involving multi-electron transfer and the generation of complex intermediates, including nitrogen-containing species with oxidation states ranging from +5 to −3. The final thermodynamically stable products are mainly N_2_ and NH_3_. Although NH_3_ is the desired product, nitrate reduction can also produce N_2_ as a major competing product. The synthesis of NH_3_ requires an eight-electron transfer, whereas the formation of N_2_ involves only a five-electron transfer. More importantly, the standard reduction potential for N_2_ formation (E = 1.17 V vs. SHE) is substantially more positive than that for NH_3_ formation (E = −0.12 V vs. SHE), indicating that the reduction of nitrate to N_2_ is thermodynamically more favorable than its reduction to NH_3_. Consequently, N_2_ is the primary competitive byproduct during the eNO_3_^−^RR. Therefore, suppressing N_2_ formation while steering the reaction pathway toward NH_3_ is one of the central challenges in the design of highly selective electrocatalysts. The reaction processes are shown below [23,24]:

$$2\;{{\mathrm{NO}}_{3}}^{-}\;+\;12\;H^{+}\;+\;10\;e^{-}\;\to \;N_{2\;}+\;6H_{2}O$$

(1) E=1.17 V vs. SHE 

$${{\mathrm{NO}}_{3}}^{-}+9\;H^{+}+8\;e^{-}\;\to \;{\mathrm{NH}}_{3}+3\;H_{2}O$$

(2) E=−0.12 V vs. SHE

During the eNO_3_^−^RR process, because N_2_ formation is thermodynamically favored, suppressing the generation of competing byproducts such as N_2_, N_2_O, and NO_2_^−^ from the environment or the reaction, to maximize the yield of NH_3_, it is also necessary to clarify the reduction mechanisms for the formation of different products. These mechanisms can be divided into indirect and direct mechanisms [25,26].

The indirect mechanism occurs only in highly acidic media. When the concentrations of NO_3_^−^ and H^+^ are greater than 1.0 M, nitrate is converted to nitric acid (HNO_3_) during the reaction [27]. However, nitrate ions do not directly participate in the electron transfer process but are reduced to generate active species such as NO^+^ and NO_2_ [26,27,28]. Based on NO_2_ or NO^+^, the autocatalytic process can be divided into two pathways: the Vetter process and the Schmid process.

In the Vetter process, NO_2_ is first reduced to nitrite (NO_2_^−^) (Reaction 3), which then undergoes protonation in a strongly acidic environment to form nitrous acid (HNO_2_) (Reaction 4) [29,30]. Subsequently, HNO_2_ and HNO_3_ react to form dinitrogen tetroxide (N_2_O_4_), which decomposes into two NO_2_ molecules (Reactions 5 and 6). In the Schmid mechanism, NO^+^ generated from the decomposition of HNO_2_ (Reaction 7) first captures an electron to form NO (Reaction 8), followed by a direct reaction between NO and HNO_3_ to produce HNO_2_ (Reaction 9), while the N_2_O_4_ generated in Reaction 5 also reacts with NO to form HNO_2_ (Reaction 10).

(3) $$NO_{2}+e^{-}\;\to \;{{\mathrm{NO}}_{2}}^{-}$$

(4) $${{\mathrm{NO}}_{2}}^{-}+H^{+}\;\to \;\mathrm{HN}O_{2}$$

(5) $$\mathrm{HN}O_{2}+\mathrm{HN}O_{3}\;\to \;N_{2}O_{4}\;$$

(6) $$N_{2}O_{4}\;\to \;2\;NO_{2}$$

(7) $$\mathrm{HN}O_{2}+H^{+}\;\to \;{\mathrm{NO}}^{+}+H_{2}O$$

(8) $${\mathrm{NO}}^{+}+e^{-}\;\to \;\mathrm{NO}$$

(9) $$\mathrm{HN}O_{3}+2\;\mathrm{NO}+H_{2}O\;\to \;3\;\mathrm{HN}O_{2}$$

(10) $$N_{2}O_{4}+2\;\mathrm{NO}+2\;H_{2}O\;\to \;4\;\mathrm{HN}O_{2}$$

In the direct mechanism, the reduction process of nitrate mainly includes the adsorption and diffusion of reactants, charge transfer, and desorption of products [31]. The transport rate of nitrate can be enhanced by an electric field [32]. In this process, the adsorption of nitrate onto active sites is the first step of the electrochemical reaction (Reaction 11) [32].

(11) $${{\mathrm{NO}}_{3}}^{-}+\;*\;\to \;*{{\mathrm{NO}}_{3}}^{-}$$

Based on *NO_3_^−^, the subsequent electrochemical reduction pathways mainly include the adsorption of *H and the electron transfer from the cathode [33]. Under the mediation of adsorbed *H, firstly, adsorbed *H is generated through the hydrogen evolution reaction (HER) during the Volmer process, and then *H can reduce *NO_3_^−^ to NH_3_. It should be noted that two *N atoms can combine to form N_2_ [34]. However, the formation of N–H bonds is energetically more favorable than the formation of N–N bonds. This suggests that an appropriate surface-*H coverage can promote NH_3_ formation, which is particularly significant in the catalytic activity of transition metal-based electrocatalysts such as Pd and Cu. During the electron transfer process, electrons from the electrode first reduce the adsorbed *NO_3_^−^ to *NO_3_^2−^ (Reaction 12), which is generally considered the rate-determining step. In this process, the transfer of electrons to the lowest unoccupied molecular orbital of *NO_3_^−^ requires a high energy barrier, but this can be improved by choosing appropriate electrode materials. As a highly oxidizing species, *NO_3_^2−^ can then be reduced to *NO_2_^−^ (Reaction 13). Subsequently, *NO_2_^−^ further accepts one electron to form *NO_2_^2−^. Finally, *NO_2_^2−^ is protonated to generate *NO (Reactions 14 and 15).

(12) $${{\mathrm{NO}}_{3}}^{-}+e^{-}\;\to \;*{{\mathrm{NO}}_{3}}^{2-}$$

(13) $${{\mathrm{NO}}_{3}}^{2-}+2\;H^{+}+e^{-}\;\to \;*{{\mathrm{NO}}_{2}}^{-}+H_{2}O$$

(14) $$*{{\mathrm{NO}}_{2}}^{-}\;+e^{-}\;\to \;*{{\mathrm{NO}}_{2}}^{2-}$$

(15) $$*{{\mathrm{NO}}_{2}}^{2-}+2\;H^{+}\;\to \;*\mathrm{NO}+H_{2}O$$

*NO is a crucial intermediate, and its adsorption behavior determines the subsequent reaction pathway and corresponding products. If *NO is adsorbed via the N-end, it tends to interact with protons and electrons to form *HNO (Reaction 16). *HNO then undergoes two consecutive protonation steps to ultimately generate NH_3_ (Reactions 17 and 18). After that, the generated *O can be further reduced to water (H_2_O). Among these electrochemical steps, the protonation of *NO to form *HNO is considered the rate-determining step.

(16) $$*\mathrm{NO}+H^{+}+e^{-}\;\to \;*H\mathrm{NO}$$

(17) $$*H\mathrm{NO}+\;H^{+}+e^{-}\;\to \;*H_{2}\mathrm{NO}$$

(18) $$*H_{2}\mathrm{NO}+\;H^{+}+e^{-}\;\to \;{\mathrm{NH}}_{3}+*O$$

If *NO is adsorbed via the O-end, the subsequent reaction depends on the N–N bond formation between *NO and solvated nitrogen oxides (NO(aq)) [35]. If an N–N bond forms between *NO and NO(aq), the intermediate can undergo subsequent proton-coupled electron transfer, leading to the formation of *HN_2_O_2_ (Reaction 19). Subsequently, *HN_2_O_2_ undergoes proton-coupled reduction to generate *N_2_O (Reaction 20). If the adsorption of *N_2_O is weak, N_2_O is preferentially generated. Otherwise, similar to the reduction pathway of *NO_3_^−^ and *NO_2_^−^, the adsorbed *N_2_O is further reduced to *N_2_O^−^ (Reaction 21) and forms N_2_ and H_2_O through protonation (Reaction 22). If N–N dimerization does not occur between *NO and solvated NO(aq), *NO will be further reduced to *NOH (Reaction 23) [35]. Subsequently, *NOH undergoes further protonation to generate *N and H_2_O (Reaction 24). Finally, *N generates NH_3_ through three consecutive direct protonation processes (Reactions 25–27).

(19) $$*\mathrm{NO}+\;{\mathrm{NO}}_{\left(\mathrm{aq}\right)}{+H}^{+}+e^{-}\;\to \;*{\mathrm{HN}}_{2}O_{2}$$

(20) $$*{\mathrm{HN}}_{2}O_{2}\;{+H}^{+}+e^{-}\;\to \;*N_{2}O+H_{2}O$$

(21) $$*N_{2}O+e^{-}\;\to \;*{N_{2}O}^{-}$$

(22) $$*{N_{2}O}^{-}\;{+2\;H}^{+}+e^{-}\;\to \;N_{2}+H_{2}O$$

(23) $$*\mathrm{NO}\;{+H}^{+}+e^{-}\;\to \;*\mathrm{NOH}\;$$

(24) $$*\mathrm{NOH}\;{+H}^{+}+e^{-}\;\to \;*N+H_{2}O$$

(25) $$*N\;{+H}^{+}+e^{-}\;\to \;*\mathrm{NH}$$

(26) $$\mathrm{NH}\;{+H}^{+}+e^{-}\;\to \;*{\mathrm{NH}}_{2}$$

(27) $${\mathrm{NH}}_{2}\;{+H}^{+}+e^{-}\;\to \;*{+\mathrm{NH}}_{3}$$

If *NO is adsorbed in a parallel manner, the protonation site will determine the subsequent reaction pathway and corresponding products [36]. If protonation occurs on the N atom, the further reaction will follow the reduction pathway under N-end adsorption. Otherwise, the intermediate *NOH will form, which undergoes further protonation on the O or N atom to generate *N or *HNOH, respectively (Reaction 28). *HNOH undergoes a two-step protonation process to produce NH_3_ (Reactions 29 and 30).

(28) $$*\mathrm{NOH}\;{+H}^{+}+e^{-}\;\to \;*H\mathrm{NOH}\;$$

(29) $$*H\mathrm{NOH}\;{+H}^{+}+e^{-}\;\to \;*H_{2}\mathrm{NOH}$$

(30) $$*H_{2}\mathrm{NOH}\;{+2\;H}^{+}+2\;e^{-}\;\to \;*\;{+\mathrm{NH}}_{3}+H_{2}O$$

The electrocatalytic reduction of NO_3_^−^ to NH_3_ involves a multi-electron transfer process. Therefore, to eliminate potential interference from the electrocatalyst itself or the external environment, the products must be quantified and their nitrogen source verified. Methods for NH_3_ detection include UV–Vis spectrophotometry (e.g., Nessler’s reagent method and indophenol blue method), ion chromatography, fluorescence methods, ammonia ion-selective electrode methods, and nuclear magnetic resonance (NMR) spectroscopy [37,38,39,40,41]. These methods show high accuracy over a wide range of ammonia concentrations. Among them, UV–Vis spectrophotometry remains the most widely used method because of its simplicity, low cost, and convenient operation. Ion chromatography provides high quantitative accuracy but generally requires sample pretreatment to eliminate interference from heavy metal ions and organic species. NMR spectroscopy possesses the unique capability to distinguish isotopically labeled nitrogen species and is therefore particularly valuable for tracing the origin of NH_3_. Particularly, ^15^N isotope-labeling experiments coupled with 1H-NMR analysis are widely recognized as the gold standard for unequivocally confirming that the produced NH_3_ originates from nitrate rather than airborne nitrogen contaminants, catalyst-derived nitrogen species, or impurities in the electrolyte. Although this method requires relatively high NH_3_ concentrations and appropriate internal standards for quantitative analysis, it provides the most reliable evidence for evaluating the intrinsic catalytic activity and NH_3_ selectivity of eNO_3_^−^RR electrocatalysts. Therefore, ^15^N isotope labeling should be considered an essential verification method for evaluating the intrinsic catalytic performance and NH_3_ selectivity of eNO_3_^−^RR electrocatalysts.

## 3. Types of Cathode Materials for Electrocatalytic Nitrate Reduction to Ammonia

The removal of nitrate is closely related to the adsorption strength of the catalyst towards N and O [42]. Generally, strong adsorption of N and O by the catalyst, in the presence of competing HER, reduces the selectivity for NH_3_ generation. Transition metal-based catalysts have garnered extensive attention in the study of eNO_3_^−^RR to NH_3_ due to their unique electronic structures and abundant reserves (Table 1). We review the catalysts used in the field of eNO_3_^−^RR to NH_3_, including monometallic catalysts, bimetallic catalysts, metal oxides, metal phosphides, metal sulfides, metal nitrides, and non-metallic catalysts.

### 3.1. Monometallic Catalysts

Owing to their structural simplicity, ready availability, and ease of preparation, monometallic electrodes have attracted considerable attention in electrocatalysis. In particular, the catalytic behavior of monometallic catalysts in eNO_3_^−^RR and the methods to improve NH_3_ selectivity are particularly noteworthy.

Goldsmith et al. systematically investigated the relationship between adsorbed species on metal surfaces such as Fe, Co, Rh, Cu, Ag, Au, Pt, and Pd and their catalytic behavior under acidic conditions [60]. Based on density functional theory (DFT) calculations and microkinetic simulations, they found that the adsorption energies of N and O atoms could serve as indicators for evaluating the selectivity of eNO_3_^−^RR. Strong N binding on catalysts such as Fe and Co tends to generate N_2_, while the formation of NO and NH_3_ requires moderate adsorption (as shown for Rh). However, due to the limited applicability of these indicators, they are not suitable for all experimental systems. For example, under acidic conditions, Rh/C (mass fraction: 10% to 20%) exhibited low nitrate conversion and NH_3_ selectivity [61]. Clark et al. further found that the performance of Rh in eNO_3_^−^RR is closely related to pH. At low pH, the adsorption and dissociation processes of HNO_2_ and NO_2_^−^ on the Rh electrode are inhibited, leading to reduced catalytic activity [62]. Therefore, we summarize the performance studies of monometallic catalysts in eNO_3_^−^RR. Investigations of various noble metal electrodes have revealed that Ru and Pd exhibit excellent catalytic activity and high selectivity.

#### 3.1.1. Ruthenium (Ru)

**Figure 1:**
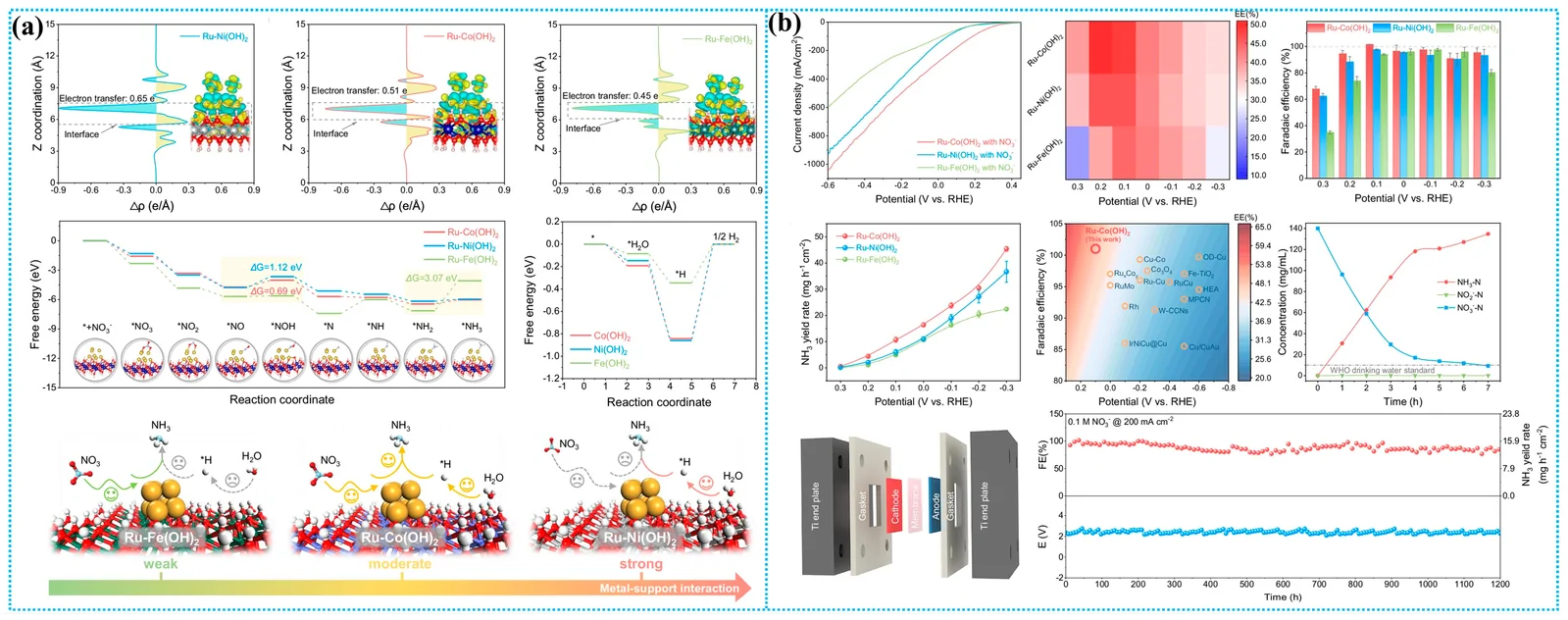
(**a**) Metal–support interaction investigation, (**b**) Electrochemical NO_3_^−^RR performance tests Adapted from Ref. [63].

Experimental and theoretical studies have examined Ru-catalyzed eNO_3_^−^RR, finding that NO_3_^−^ can be stably adsorbed on the Ru surface and converted to NH_3_ via the eNO_3_^−^RR process (Figure 1a,b) [63]. Urakawa et al. experimentally demonstrated that Ru/C exhibits high activity for nitrate conversion to NH_3_. By regulating the proton transport to suppress the competing HER, they achieved a faradaic efficiency (FE) for NH_3_ as high as 94% [61].

Structural engineering is an effective strategy to enhance the catalytic performance of Ru. For example, Yu et al. found that the catalytic activity of Ru clusters was significantly improved by applying strain [64]. The enhanced catalytic activity was attributed to the rapid generation of *H. Under a low kinetic barrier, *H further promotes the conversion of nitrate to NH_3_ through the hydrogenation rate-determining step intermediates. The results showed that at a current density of 120 mA cm^−2^, the strained Ru nanostructures maintained nearly 100% NH_3_ selectivity for 100 h due to the stable coordination of Ru–O.

**Figure 2:**
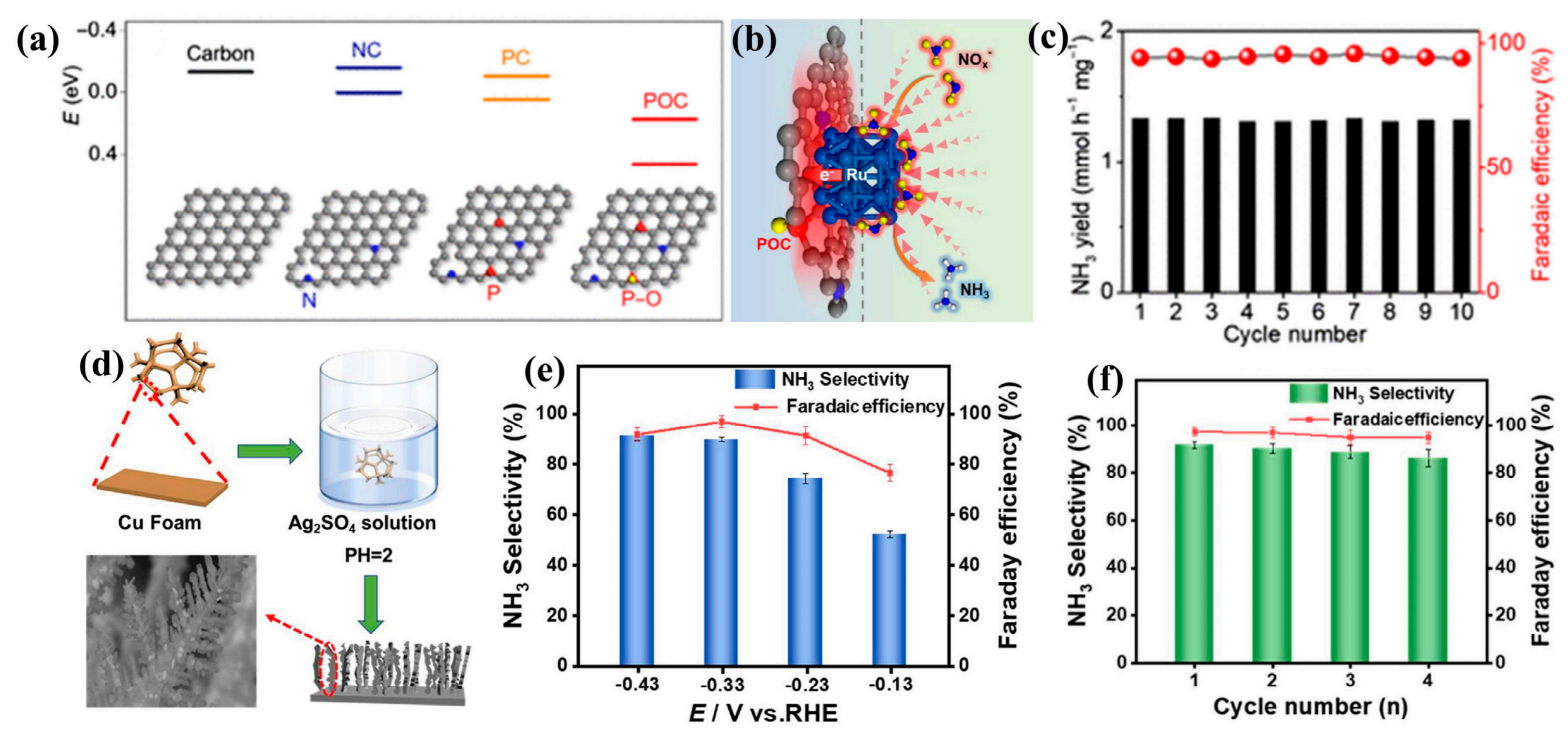
(**a**) The calculated band structure of carbon, carbon material doped with N atoms (NC), carbon material doped with P atoms (PC), and POC via DFT calculation methods; (**b**) eNO_3_^−^RR diagram of Ru/POC; (**c**) the cycling performance of the Ru/POC-based electrode at −0.8 V versus RHE [43]. (**d**) Preparation of the nano-Ag catalyst; (**e**) Selectivity and Faradaic efficiency of the reduction of nitrate to ammonia by nano-Ag at different potentials; (**f**) Stability test at −0.33 V vs. RHE over nano-Ag cathode (4 cycles). Adapted from Ref. [47].

Tuning crystallinity is also an effective way to improve catalytic performance. Research by Jin et al. showed that ultrafine amorphous Ru nanoclusters grown in situ on carbon nanotubes can serve as efficient eNO_3_^−^RR electrocatalysts [65]. The strong d–π interaction between Ru and the carbon nanotubes leads to a uniform distribution of ultrafine Ru nanoclusters and excellent long-term stability. Compared with crystalline Ru, amorphous Ru nanoclusters rich in low-coordination atoms can provide more catalytically active sites. Consequently, amorphous Ru nanoclusters (a_1_-Ru/CNTs) exhibited a high NH_3_ yield of 145.1 μg h^−1^ mg^−1^ and an FE of 80.62% at −0.2 V vs. RHE. Additionally, Chen et al. synthesized an amorphous material loaded with Ru nanoparticles (Ru/POC) by introducing P–O groups (POC) onto a carbon support and embedding Ru. Experimental results showed that under neutral conditions, Ru/POC significantly enhanced nitrate adsorption capacity and reduced the free energy difference (Figure 2a,b). Cycling stability tests at −0.8 V vs. RHE revealed a stable NH_3_ yield of 1.336 mmol h^−1^ mg^−1^ and an FE as high as 96% (Figure 2c) [43].

#### 3.1.2. Palladium (Pd)

Palladium-based nanocatalysts exhibit excellent activity in eNO_3_^−^RR, kinetically attributed to fast proton coupling and electron transfer. However, studies have found that Pd on non-reducible supports cannot effectively reduce nitrate [66,67]. Conversely, Pd supported on reducible metal oxides such as SnO_2_ and CeO_2_ shows significant catalytic activity during eNO_3_^−^RR [68]. Therefore, providing co-catalysts can influence nitrate adsorption, thereby accelerating its conversion to NH_3_ [69]. For example, Jin et al. reported the catalytic behavior of Pd nanocluster-modified Zr-MOF (PdNDs/Zr-MOF) in a 0.1 M Na_2_SO_4_ + 500 ppm NO_3_^−^ electrolyte solution. At −1.3 V vs. RHE, a high NH_3_ yield of 287.31 mmol h^−1^ g^−1^ and an FE of 58.1% were achieved, with Zr-MOF serving as an additional co-catalyst [44]. Furthermore, other non-metallic reducible supports such as BC_2_N can also promote nitrate adsorption and NH_3_ synthesis on Pd-based catalysts [70]. DFT calculations indicate that nanoscale Pd supported on boron carbonitride (BCN) possesses strong catalytic activity for the electrochemical reduction of nitrate. Hu et al. further synthesized the electrocatalyst BC_2_N/Pd (with B and N loadings both >20%) by modifying nanoscale Pd on a BCN support. This material was prepared on a stable supramolecular assembly formed by cucurbituril, aiming to in situ reduce Pd^2+^ to Pd nanoclusters. The results showed that in an alkaline nitrate electrolyte solution, the BC_2_N/Pd electrocatalyst achieved an NH_3_ yield of 1730 μg h^−1^ cm^−2^ and an FE of 70.47% for nitrate reduction at −0.7 V vs. RHE [70].

Facet engineering is an effective strategy for improving eNO_3_^−^RR performance because different crystallographic surfaces can regulate the adsorption and conversion of nitrate-derived intermediates, thereby altering the reaction pathway and product selectivity [71]. For instance, the Hatzell research team controlled the shape and exposed facets of Pd nanoparticles, designing various structures, including Pd nanocubes (six (100) facets), octahedra (eight (111) facets), concave nanocubes ((100) facets and high-index facets), cuboctahedra (combining six (100) and eight (111) facets), and polycrystalline Pd (mixed (111), (200), (220), and (311) facets) [72]. It was found that the Pd(111) facet can selectively reduce eNO_3_^−^ to NO_2_^−^, attributed to the rapid dissociation of *NO_3_^−^, while the Pd(100) facet can selectively reduce NO_2_^−^ to NH_3_ due to its more stable adsorption of NO_2_^−^. This synergistic effect makes Pd cuboctahedra exhibit a high NH_3_ yield. Zhou et al. prepared a Pd/NF catalyst by loading self-supported Pd arrays on nickel foam (NF), providing numerous active sites for *H adsorption and NO_3_^−^ activation. At −1.4 V vs. RHE, this catalyst exhibited an excellent NH_3_ yield of 1.52 mmol h^−1^ cm^−2^ with an FE of 78%. Notably, in a membrane-free flow electrolyzer, a large-area electrode of 5 × 5 cm^2^ achieved an NH_3_ yield of 0.41 mmol cm^−2^ h^−1^ at 8 V. Furthermore, the catalyst maintained a high NH_3_ yield during 25 h of testing, demonstrating good cycling stability [46].

Although these studies demonstrate the considerable potential of flow electrolyzers and large-area electrodes for practical nitrate electroreduction, most reported performances are still evaluated under laboratory-scale half-cell conditions. The direct translation of these results to industrial full-cell electrolyzers remains challenging. In practical continuous-flow systems, electrolyte circulation can significantly influence reactant transport, local pH, and catalyst stability, leading to performance deviations from those observed in static laboratory tests. Additionally, because NH_3_ is generally produced in relatively dilute electrolytes, efficient product separation and purification remain important challenges for reducing the overall process cost. Therefore, besides NH_3_ yield and Faradaic efficiency, future studies should also evaluate full-cell performance using practical metrics such as energy efficiency and specific energy consumption (kWh kg^−1^ NH_3_), thereby providing a more comprehensive assessment of the industrial feasibility of eNO_3_^−^RR systems. These results demonstrate the considerable potential of Pd-based catalysts for large-scale nitrate electroreduction. Nevertheless, several challenges remain before laboratory-scale half-cell performance can be translated into practical industrial electrolyzers.

#### 3.1.3. Other Noble Metals

Research on other noble metals in eNO_3_^−^RR is also progressing [73]. For example, Li et al. found that nano-Ag can achieve highly selective eNO_3_^−^RR (Figure 2d). At −0.33 V vs. RHE, the current density for NH_3_ reached −194.31 mA cm^−2^, with an FE as high as 96.4% and a selectivity of 89.6%. Additionally, nano-Ag demonstrated excellent stability, retaining 91.1% of its original selectivity and nearly 100% FE after four cycles, indicating its high efficiency as an electrocatalyst for NO_3_^−^ reduction to NH_3_ (Figure 2e,f) [47]. Chen et al. successfully synthesized hollow iridium nanotubes (Ir NTs) with a rough porous surface using a 1-hydroxyethylidene-1,1-diphosphonic acid-induced self-template method under hydrothermal conditions. They studied their electrocatalytic performance for NO_3_^−^ reduction to NH_3_ in acidic electrolyte [48]. The unique one-dimensional porous structure endowed Ir NTs with a large surface area, high conductivity, and superior atomic utilization efficiency, achieving an NH_3_ yield of 921 μmol h^−1^ mg^−1^ and an FE of 84.7% at 0.06 V vs. RHE. However, the high cost and scarcity of noble metals limit their large-scale application. Therefore, researchers have begun exploring inexpensive transition metals as alternatives. Among the many transition metals, Cu and Co have shown the highest NH_3_ yields and best selectivity.

#### 3.1.4. Copper (Cu)

**Figure 3:**
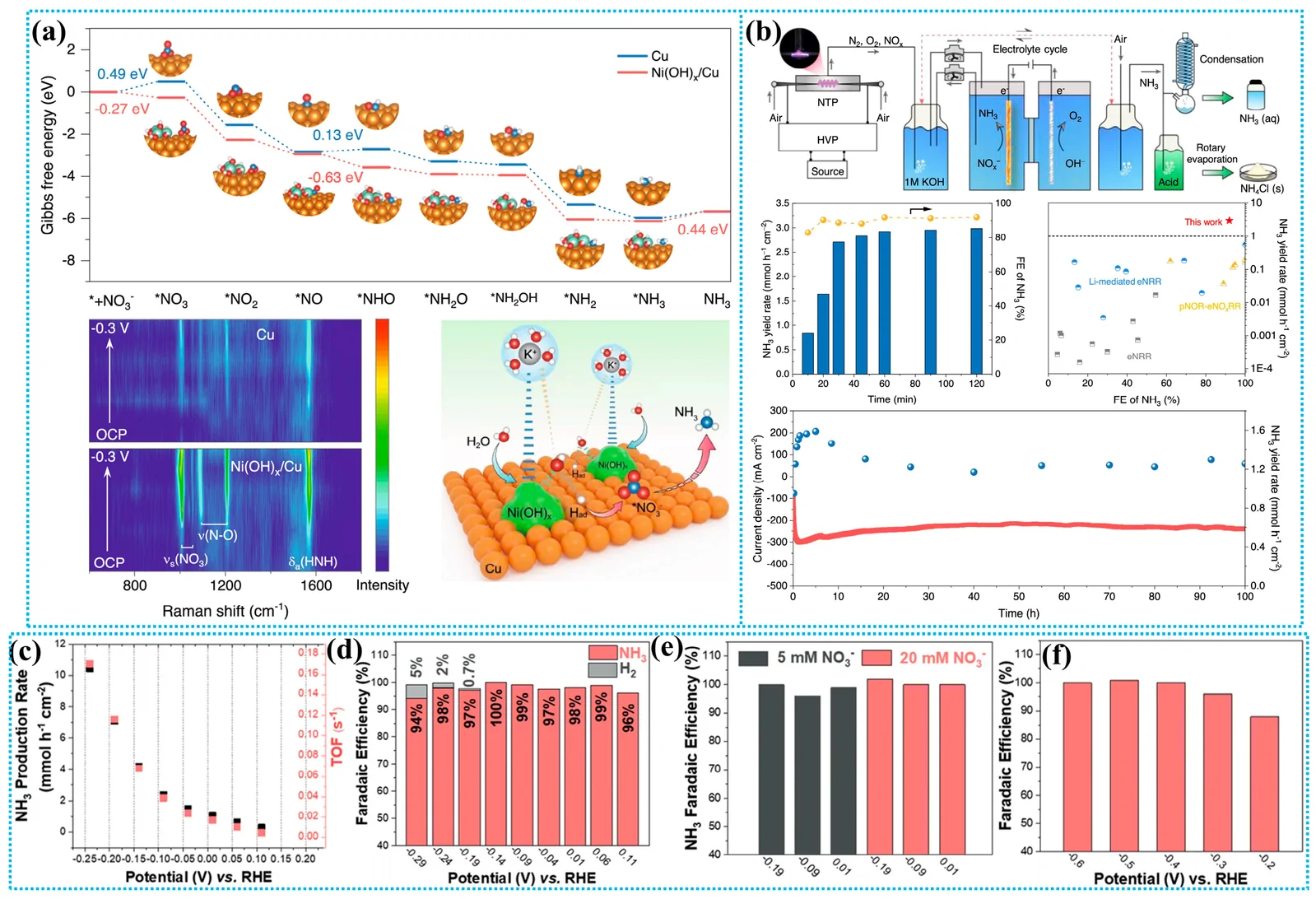
(**a**) Investigation of the eNO_x_^−^RR mechanism. (**b**) Performance of pNOR-eNO_x_^−^RR system for air-to-NH_3_ conversion [74]. (**c**) Calculated NH_3_ production rate and TOF from NITRR catalyzed by Co-NAs by assuming all Co atoms are catalytically active. (**d**) Potential-dependent Faradaic efficiency of Co-NAs toward NH_3_ and H_2_. (**e**) Faradaic efficiency toward NH_3_ production in 1 m KOH containing 5 and 20 mm NO_3_^−^. (**f**) Faradaic efficiency toward NH_3_ production from −0.2 to −0.6 V in 0.5 m K_2_SO_4_ containing 100 mm NO_3_^−^. Adapted from Ref. [82].

Theoretical and experimental studies indicate that Cu-based catalysts follow a direct reduction mechanism in eNO_3_^−^RR, where the rate-determining step “*NO to *NOH” has a low limiting potential, leading to their good catalytic performance (Figure 3a,b) [74]. Compared with Ru and Pd, Cu is not only inexpensive and abundant but also widely used in industry. For example, commercial Cu foam has been used as an electrocatalyst for eNO_3_^−^RR [75]. Although its selectivity for NH_3_ is as high as 82.5%, slow kinetics reduce nitrate conversion and FE. To enhance catalytic performance, researchers have investigated the structural effects of Cu computationally and experimentally [76,77,78,79]. Guo et al. found that the theoretical activities of Cu(100) and Cu(111) in eNO_3_^−^RR are higher than those of Cu(110), with Cu–Cu dimers of specific bond lengths considered active species in the reaction pathway. Additionally, Cu(100) is active over a wide pH range (1.42–14.0), while Cu(111) exhibits optimal activity in neutral or alkaline environments [79]. However, considering solvent effects, Shao et al. reported a different ordering of facet activity [78]. Using implicit and explicit solvent models to simulate the H_2_O/Cu interface under pH control, they found that the Cu(100) facet is more favorable for NH_3_ synthesis than the Cu(111) facet, especially under high pH conditions, due to its lower thermodynamic and kinetic barriers. Furthermore, their experiments demonstrated that the reaction pathway is independent of pH [76,77].

Copper nanoribbons rich in Cu(100) (Cu-NB-100) exhibited superior activity in eNO_3_^−^RR compared to those rich in Cu(111) (Cu-NB-111). At −0.15 V vs. RHE, Cu-NB-100 achieved an NH_3_ yield as high as 650 mmol g^−1^ h^−1^ (>350 mA cm^−2^) with an FE exceeding 95% [76]. Wang et al. further verified through experiments and DFT calculations that the Cu(100) facet is more active than the Cu(111) facet [77]. Therefore, selecting an appropriate solvent model is crucial for accurately predicting catalytic behavior. Moreover, the crystallinity and surface morphology of Cu also significantly affect its catalytic performance in eNO_3_^−^RR. For example, Gao et al. found that a Cu-based catalyst with a flower-like clustered structure exhibited excellent eNO_3_^−^RR performance at −0.266 V vs. RHE, with an NH_3_ yield of 101.4 μmol h^−1^ cm^−2^ and an FE of 93.91%. This was mainly attributed to the abundant active sites provided by the Cu(100) and Cu(111) facets and their synergistic effect [80]. Hu et al. showed that amorphous Cu/PCNF exhibited higher NH_3_ yield and FE than its crystalline counterpart, because amorphous Cu/PCNF enhanced nitrate adsorption and promoted the protonation of *NO to *NHO [49]. Additionally, amorphization also improved the stability of eNO_3_^−^RR during long-term operation, especially under high negative potentials. The support substrate and lattice strain also significantly affect catalytic performance. For example, Guo et al. electrodeposited Cu on Ni foam, Cu foam, and carbon cloth, finding that Cu on Ni foam exhibited the best catalytic performance, mainly because Ni foam could promote *H adsorption, thereby accelerating NH_3_ generation [81]. Liu et al. reported that lattice strain induced by stacking faults could accelerate NO_3_^−^ adsorption and inhibit H_2_ generation, thereby improving the reduction of nitrate to NH_3_ [50]. These studies indicate that rational selection of support substrates and modulation of lattice strain are effective strategies to enhance catalyst performance.

#### 3.1.5. Cobalt (Co)

Although Co does not show significant selectivity for NH_3_ generation via eNO_3_^−^RR in acidic electrolyte solutions [60], some experimental results under alkaline conditions indicate it has high NH_3_ yields and good selectivity [82]. For instance, Luo et al. prepared metallic cobalt nanoarrays (Co NAs) by electrochemical reduction of Co(OH)_2_ nanoarrays, which exhibited excellent eNO_3_^−^RR performance for NH_3_ production. At −0.24 V vs. RHE, Co NAs achieved an NH_3_ yield of 10.4 mmol h^−1^ cm^−2^ in 1 M KOH + 0.1 M KNO_3_ electrolyte, attributed to adsorbed nitrogen-containing species accelerating H_2_O dissociation and surface-*H generation (Figure 3c). Moreover, a near-unity Faradaic efficiency (FE) for NH_3_ production (≥96%) is maintained over a broad operational window, including a wide range of applied potentials, NO_3_^−^ concentrations, and pH values (Figure 3d–f) [82]. Singh et al. confirmed the superior activity of the Co(100) facet in eNO_3_^−^RR via DFT calculations [83]. Additionally, they found that Co-derived oxides act as efficient eNO_3_^−^RR catalysts, achieving an NH_3_ partial current density of 565.26 mA cm^−2^ and an FE up to 92.37% at −0.8 V vs. RHE [83]. Wang et al. prepared dense film-like Co nanomaterials (DM-Co) using a combination of chemical and electrochemical deposition, finding an NH_3_ yield of 1.2 mmol h^−1^ cm^−2^ and an FE close to 100% at −0.2 V vs. RHE [84].

#### 3.1.6. Other Transition Metals

Jaramillo et al. found that high selectivity for eNO_3_^−^RR could only be achieved with high concentrations of protons and nitrate ions. In an electrolyte containing 0.4 M NO_3_^−^ at pH 0.77, an NH_3_ partial current density of −22 mA cm^−2^ and a maximum FE of 82% were achieved at −1.0 V vs. RHE [85]. However, other transition metals such as Ni [73], Bi [86], and Sn [87] did not show significant NH_3_ yields due to low selectivity and high overpotentials. Subsequently, the Stoerzinger group combined calculations and experiments to study the eNO_3_^−^RR behavior of different transition metal catalysts in neutral media, building microkinetic models to demonstrate that competitive adsorption between NO_3_^−^ and *H is the root cause of differences in NH_3_ selectivity [88]. Therefore, transition metals with high HER activity (e.g., Ni) show significantly reduced selectivity for NH_3_ via eNO_3_^−^RR. Furthermore, they found that NH_3_ selectivity could be improved when the d-band center of the metal was closer to its Fermi level. However, among materials with similar d-band center energies, Co exhibited the best catalytic performance due to its strong adsorption of NO_2_^−^, which promotes NO dissociation and enhances the efficiency of *N reduction to NH_3_. Wen et al. used Ni nanoparticles with grain boundary (GBs) defects for electrocatalytic NO_3_^−^ reduction, achieving an NH_3_ yield as high as 15.49 mmol h^−1^ cm^−2^ and an FE of 93% at −0.93 V vs. RHE. This performance exceeded that of the comparison materials, including Cu and noble metals. This is because GBs regulate *H, causing it to be consumed in the eNO_3_^−^RR pathway rather than forming H_2_, thereby significantly suppressing HER while promoting *NO_3_^−^ adsorption and effectively enhancing the conversion rate from *NO_3_^−^ to *NO_2_^−^, the rate-determining step [51].

From the overview above, it is evident that the eNO_3_^−^RR performance of monometallic catalysts can be significantly improved through strategies such as morphology control, crystallinity regulation, and co-catalyst selection [89]. Although significant progress has been made in the study of monometallic eNO_3_^−^RR, their NH_3_ yields are still relatively low to meet industrial application requirements. Additionally, the high cost and insufficient stability of monometallic catalysts greatly limit their commercial application. To address these issues, researchers have begun considering the use of metal alloys to improve eNO_3_^−^RR performance.

### 3.2. Bimetallic Catalysts

Due to their tunable electronic structures, good stability, and lower cost, alloys have been widely investigated in various catalytic reactions, such as H_2_O decomposition, eNRR and eCO_2_RR [90,91,92]. Therefore, researchers have extensively studied the behavior of bimetallic alloys in eNO_3_^−^RR.

#### 3.2.1. Ruthenium-Based Alloys

The catalytic behavior of Ru-based alloys in eNO_3_^−^RR has been systematically elucidated through theoretical simulations and experimental studies. In 2019, the Goldsmith team revealed through DFT calculations that Pt_3_Ru alloys possess both high catalytic activity and selectivity in eNO_3_^−^RR [60]. Further experimental studies showed that adjusting the Ru content in Pt_x_Ru_y_ alloys supported on carbon felt (Pt_x_Ru_y_/CF) in acidic media could optimize the adsorption energies of nitrate and *H and regulate the reaction limiting potential. When the Ru content was 37%, Pt_63_Ru_37_/CF exhibited optimal eNO_3_^−^RR catalytic performance [19]. Yu et al. recently designed a series of Ru_x_Co_y_ hollow nanodecahedra (HNDs) and found that Ru_15_Co_85_ HNDs achieved NH_3_ yields of 3.21 ± 0.17 mol g^−1^ h^−1^ and an FE of 97% at 0 V vs. RHE [52]. This phenomenon is attributed to Ru sites accelerating the conversion kinetics of NO_2_^−^ to NH_3_, while the electrocatalytic reduction of cobalt hydroxide (Co(OH)_2_) to metallic Co further optimizes the reaction pathway. Based on a similar mechanism, Ru_15_Ni_85_ and Ru_15_Fe_85_ HNDs also exhibited significant NH_3_ synthesis efficiency.

**Figure 4:**
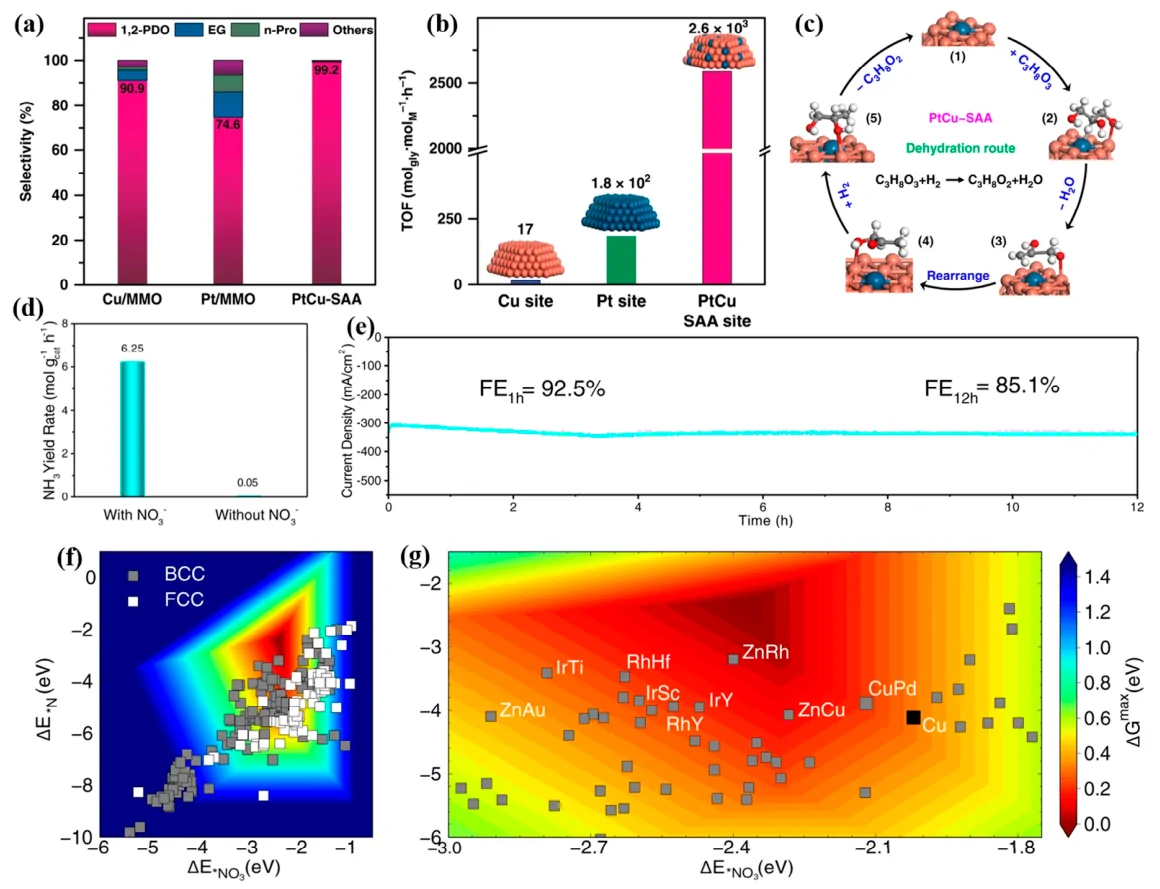
Catalytic evaluation of PtCu-SAA and monometallic catalysts (Pt/MMO and Cu/MMO) toward glycerol hydrogenolysis to 1,2-PDO: (**a**) the product selectivity, (**b**) the TOF value determined as moles of initial glycerol converted per mole of exposed active sites per hour in the catalytic dynamic range, and (**c**) schematic representation for the reaction mechanism. Adapted from Ref. [95]. (**d**) The yield rate of NH_3_ with or without nitrate at −0.6 V vs. RHE. (**e**) Stability test by running the CA measurement on the ordered CuPd nanocubes at −0.5 V vs. RHE for 12 h. (**f**) DFT-calculated adsorption energies of *NO_3_ and *N on (100)-terminated B2 intermetallics from the Materials Project and randomly sampled (100)-terminated fcc intermetallics. (**g**) DFT-calculated adsorption energies of *NO_3_ and *N on (100)-terminated B2 intermetallics close to the activity volcano top Cu(100) and a few interesting systems (the first element denotes the surface metal) are highlighted. Adapted from Ref. [98].

Single-atom alloys (SAAs), which disperse trace amounts of heteroatoms into the lattice of a host metal, can modulate electronic structures and enhance catalytic performance, garnering attention in the eNO_3_^−^RR field [93,94,95,96]. Zhang et al. synthesized a PtCu single-atom alloy catalyst, which achieved a 98.8% yield of 1,2-propanediol and a turnover frequency of 2.6 × 10^3^ mol mol^−1^ h^−1^, outperforming previously reported heterogeneous metal catalysts for glycerol hydrogenolysis (Figure 4a,b). Mechanistically, isolated Pt–Cu interfacial sites act as the intrinsic active centers, where single Pt atoms promote cleavage of the central C–H bond, while adjacent Cu atoms facilitate terminal C–O bond dissociation, thereby enabling a lower-energy hydrogenolysis pathway (Figure 4c). Initially, glycerol is adsorbed and activated at the Pt–Cu interfacial sites, where the C–O bond of the primary hydroxyl group interacts preferentially with the Cu atom, while the C–H bond at the secondary carbon is activated on the adjacent Pt site. Subsequently, the cooperative activation of these bonds facilitates the dehydration step through the cleavage of the primary C–O bond and secondary C–H bond, generating a 2,3-enol intermediate. This intermediate then undergoes structural rearrangement to form acetol, which is further hydrogenated by activated H species to yield the desired 1,2-propanediol (1,2-PDO) product. The enhanced catalytic performance and high selectivity originate from the atomic-level synergistic interaction between isolated Pt atoms and Cu sites, which enables the construction of highly efficient and selective catalytic centers. [95]. The Ru single-atom dispersed Cu nanowires (Ru–Cu SAAs) developed by Muhich et al. achieved an FE of 93% at a current density of 1 A cm^−2^ [97]. Here, isolated Ru atoms selectively promote the NH_3_ generation pathway and suppress side reactions, while the Cu matrix effectively inhibits the competing HER by modulating hydrogen adsorption energy. Notably, this catalyst demonstrated over 90% FE after 100 h of cycling in a continuous flow reaction system treating industrial wastewater containing 2000 ppm NO_3_^−^ at a current density of 400 mA cm^−2^, with the effluent meeting purification standards, showcasing its potential for industrial applications.

#### 3.2.2. Palladium-Based Alloys

Research shows that CuPd or PtRu alloys possess significant electrocatalytic activity for converting nitrate to NH_3_ [98,99]. Experimental results indicated that CuPd nanocubes in alkaline media exhibited a high NH_3_ yield of 6.25 mol h^−1^ g^−1^ at −0.6 V vs. RHE and an FE of 92.5% at −0.5 V vs. RHE (Figure 4d,e). This performance enhancement is mainly attributed to enhanced *NO_3_^−^ adsorption on Cu sites and weakened *N adsorption on Pd sites (Figure 4f,g) [98].

Bi has been applied in eNRR due to its ability to activate N-containing species and inhibit HER [100,101]. Therefore, the application of Bi–Pd alloys in eNO_3_^−^RR has also attracted considerable attention. For instance, Chu et al. [54] reported bismuth–palladium metallene (Bi_1_Pd) as an efficient eNO_3_^−^RR catalyst, achieving an NH_3_ FE close to 100% and an NH_3_ yield of 33.8 mg h^−1^ cm^−2^ at −0.6 V vs. RHE, surpassing almost all reported eNO_3_^−^RR catalysts. In-depth theoretical and spectroscopic studies further revealed that single-atom Bi synergizes with adjacent Pd atoms to effectively activate NO_3_^−^ and destabilize NO on the Bi_1_Pd surface, thereby lowering the energy barrier for the key step (NO → *NOH) and enhancing the protonation ability during the NO_3_^−^ to NH_3_ conversion.

#### 3.2.3. Copper-Based Alloys

Copper-based alloys have garnered significant attention due to their low cost, high activity, and abundant earth resources, especially when alloyed with transition metals rich in d-electrons [56]. To elucidate the structure–activity relationship, the Sargent research team [56] focused on CuNi alloys with different Cu/Ni ratios. They found that Cu_50_Ni_50_ exhibited excellent eNO_3_^−^RR performance at 0 V vs. RHE, with a current density approximately six times that of pure Cu and an FE as high as 99%. Combining calculations and experimental studies, the team pointed out that Ni can modulate the d-band center of Cu, enhancing intermediate adsorption and increasing reaction activity. Based on this, the researchers proposed a correlation between adsorption energy and reaction activity, guiding for designing efficient eNO_3_^−^RR catalysts.

Other theoretical and experimental studies support this conclusion [102,103]. For example, Guo et al. found that Cu_49_Fe_1_ exhibited higher activity and selectivity in eNO_3_^−^RR, attributed to the shift in Cu’s d-band center towards the Fermi level [104]. Liang et al. conducted a comparative study on CuFe, CuCo, and CuNi alloys (Cu content = 30%, 50%, 70%) supported on ordered mesoporous carbon (Cu_x_M_y_/OMC), finding that all these alloys showed high performance in eNO_3_^−^RR. Particularly, Cu_5_Fe_5_/OMC achieved an NH_3_ yield of 365.9 μg h^−1^ mg^−1^ at −0.8 V vs. RHE [55].

Furthermore, due to synergistic effects with other metals, Cu is considered an excellent support for single-atom alloys. For instance, Zang et al. reported that Ni-alloyed Cu (Ni_1_Cu) achieved an NH_3_ yield of 326.7 μmol h^−1^ cm^−2^ at −0.55 V vs. RHE with 200 ppm NO_3_^−^, approximately 10.7 times higher than pure Cu [57]. X-ray absorption spectroscopy data and DFT calculations indicated that the shift in the d-band center of Ni atoms towards the Fermi level and the increased N 2p orbital bond energy accelerate the eNO_3_^−^RR process and inhibit H_2_ byproduct formation. Yin et al. found that a Au_1_Cu single-atom alloy with single Au atoms and Cu vacancies (VCu-Au_1_Cu) achieved an NH_3_ yield of 555 μg h^−1^ cm^−2^ and an FE of 98.7% at −0.2 V vs. RHE. Experimental and computational results showed that Au single atoms and Cu vacancies promote *H formation and NO_3_^−^ adsorption, thereby facilitating NH_3_ generation via the adsorbed hydrogen mechanism [58].

**Figure 5:**
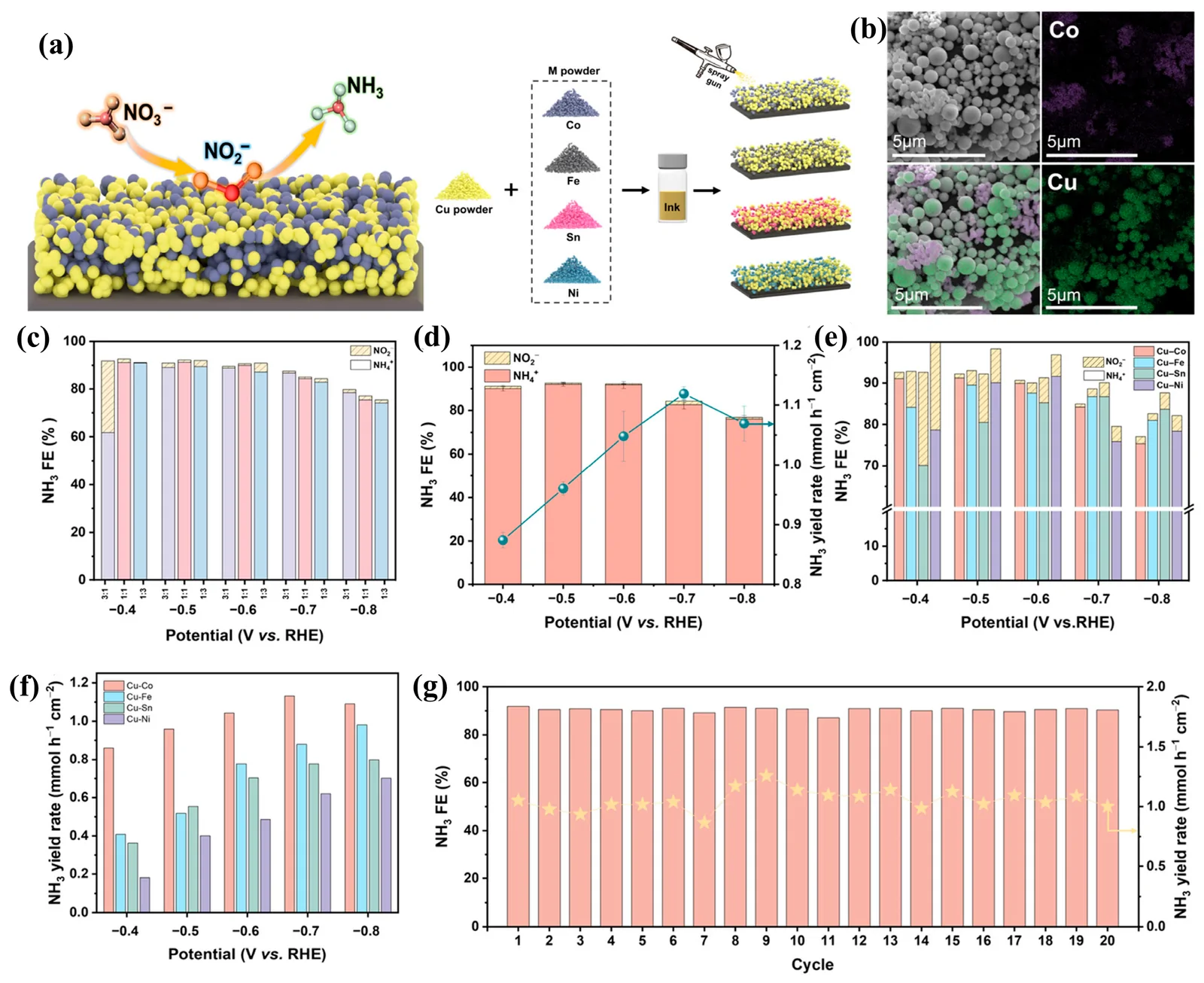
(**a**) Schematic of the spray-coating fabrication process for Cu–M catalysts. (**b**) Representative morphology and elemental distribution characterization of Cu–Co. (**c**) FE of NH_3_ and NO_2_^−^ for catalysts with different Cu: Co mass ratios (3:1, 1:1, 1:3), where the hatched and solid bars represent NO_2_^−^ and NH_3_, respectively. (**d**) Potential-dependent NH_3_ FE and NH_3_ yield rate for the optimized Cu–Co (1:1) catalyst. (**e**) Comparison of NH3 FE for Cu–M catalysts at their respective optimal compositions, where the hatched and solid bars represent NO_2_^−^ and NH_3_, respectively. (**f**) Comparison of NH_3_ yield rate for Cu–M catalysts at their respective optimal compositions. (**g**) Chronoamperometric stability test of the optimized Cu–Co (1:1) catalyst at −0.5 V (vs. RHE). Adapted from Ref. [106].

Alloys loaded with metals such as Cu are also effective eNO_3_^−^RR catalysts. Garcia-Segura et al. synthesized Cu–Pt foam electrodes by depositing Pt onto Cu foam, finding that they could promote the outer-sphere protonation of NO_2_^−^, thereby accelerating NO_3_^−^ reduction. With the optimal Cu–Pt electrode, almost all nitrate was removed from a 30 ppm NO_3_^−^ solution after 2 h of electrolysis, achieving an NH_3_ selectivity of 84% [105]. Luo et al. prepared uniformly dispersed Rh with different morphologies on Cu nanowires via a galvanic displacement reaction and found that Rh@Cu-0.6%, composed of Rh clusters and Cu single atoms, achieved an NH_3_ yield of 21.61 mg h^−1^ cm^−2^ at −0.4 V vs. RHE, significantly higher than bare Cu nanowires and Rh@Cu-12.5% [59]. Additionally, Zhang et al. systematically investigated Cu–M catalytic systems and demonstrated that Cu primarily governs NO_3_^−^ adsorption and its initial reduction to NO_2_^−^, whereas the secondary metal determines the subsequent hydrogenation of NO_2_^−^ intermediates and NH_3_ selectivity (Figure 5a,b). Cu–Co exhibited the optimal kinetic matching, achieving an NH_3_ Faradaic efficiency of 91.2%, minimal NO_2_^−^ accumulation of ≈1.0%, and an NH_3_ yield rate of 0.86 mmol h^−1^ cm^−2^, thereby identifying NO_2_^−^ hydrogenation as the key selectivity-determining step in tandem nitrate-to-ammonia conversion (Figure 5c–g). The long-term stability of the Cu–Co catalyst was assessed through successive chronoamperometric measurements at −0.5 V vs. RHE. After 20 continuous electrolysis cycles, the catalyst maintained a robust catalytic performance, with both the NH_3_ Faradaic efficiency and production rate remaining nearly unchanged and preserving over 90% of their initial values (Figure 5g) [106].

Alloying significantly improves the electronic structure of catalysts, thereby accelerating intermediate adsorption and lowering the energy of the rate-determining step, thereby accelerating the kinetic conversion of nitrate to NH_3_. Beyond conventional bimetallic alloys and heterostructures, the rational construction of bimetallic single-atom catalysts has recently emerged as a promising strategy for further enhancing the activity and selectivity of eNO_3_^−^RR by maximizing atomic utilization and precisely regulating the local electronic structure. For example, Li et al. developed a FeCu bimetallic single-atom catalyst anchored on N-doped hierarchical porous carbon spheres (FeCu-NPCS). Owing to the synergistic electronic interaction between neighboring Fe and Cu atomic sites, the catalyst exhibited an excellent NH_3_ Faradaic efficiency of 97.42% and an NH_3_ yield of 82.72 mg h^−1^ mg^−1^. Furthermore, when assembled into a Zn–NO_3_^−^ battery, the FeCu-NPCS catalyst maintained excellent electrochemical stability over 1000 charge–discharge cycles, demonstrating its considerable potential for practical ammonia synthesis and integrated energy conversion [107]. These findings indicate that incorporating dual-metal single-atom active sites is an effective strategy to simultaneously optimize the electronic structure, reaction pathway, and catalytic stability, representing an important direction for the future design of highly efficient bimetallic electrocatalysts for eNO_3_^−^RR.

### 3.3. Metal Oxide Catalysts

Transition metal oxides have been widely used in various catalytic reactions due to their high durability, ease of preparation, low cost, and tunable electronic properties [108,109,110]. To clarify the influence of oxidation state and electronic structure on catalytic performance, researchers have systematically studied crystalline systems for eNO_3_^−^RR, particularly copper oxides (CuO_x_), titanium oxides (TiO_x_), and cobalt oxides (CoO_x_).

#### 3.3.1. Copper Oxides (CuO_x_)

Owing to the favorable nitrate reduction activity of Cu, CuO_x_ has attracted considerable attention for elucidating the reduction mechanism and improving catalytic activity. The oxidation state of CuO_x_ is a key factor affecting eNO_3_^−^RR selectivity, and multiple studies have explored this in detail. Zhang et al. reported that CuO nanowire arrays achieved an FE as high as 95.8% and NH_3_ selectivity of 81.2% after 2 h of reaction in 200 ppm NO_3_^−^ at −0.85 V vs. RHE [111]. This is because the CuO nanowires were in situ reduced to Cu/Cu_2_O, thereby limiting HER and accelerating the rate-determining step. Xiao et al. conducted thermodynamic and kinetic studies on the facets, applied potential, and exposed surfaces of Cu_2_O, revealing the eNO_3_^−^RR mechanism on perfect and defective Cu_2_O surfaces [112]. The defective Cu_2_O (110) and (111) facets exhibited better catalytic performance than pure Cu facets, consistent with the results from mixed Cu/Cu_2_O nanowire arrays. Li et al. found that the catalytic activity of heterostructured CuO and Cu_2_O (CuO@Cu_2_O) was superior to that of Cu_2_O and CuO alone because NO_3_^−^ could adsorb optimally on the heterostructure, lowering the energy barrier. Cu_2_O exhibited better activity in eNO_3_^−^RR than CuO because Cu^+^ enhances NO_3_^−^ adsorption [113].

Introducing O vacancies is an effective strategy for tuning the electronic structure and improving eNO_3_^−^RR. Chen et al. found that plasma-treated O-deficient Cu_2_O showed enhanced eNO_3_^−^RR activity because O vacancies increased charge density, enhanced nitrate adsorption, and accelerated the rate-determining step “*NO to *NOH” [114]. The Amal group reported that O-deficient CuO achieved an NH_3_ yield as high as 334 μmol h^−1^ cm^−2^ at −0.7 V vs. RHE [115]. In a flow electrolyzer, CuO also demonstrated high NH_3_ yields [116,117].

Combining Cu with other elements can also effectively promote eNO_3_^−^RR [118,119,120]. Wang et al. designed and constructed Pd (2.93 at%) Cu_2_O corner-etched octahedra (Pd–Cu_2_O CEO) with cavities and O vacancy defects for selective eNO_3_^−^RR to NH_3_. The unique concave cavity structure, abundant surface O vacancies, and Pd–Cu_2_O dual active sites synergistically promoted the eNO_3_^−^RR pathway. Pd–Cu_2_O CEO achieved an NH_3_ yield of 925.11 μg h^−1^ mg^−1^ and an FE of 96.56% at −1.3 V vs. RHE [120]. The Ai Wei group at Northwestern Polytechnical University reported that Zn-doped CuO nanosheets could significantly increase NH_3_ yield [119]. Ir incorporation into CuO also effectively enhanced the eNO_3_^−^RR process [118]; the improved catalytic activity was attributed to accelerated electron transfer and enhanced reaction kinetics, while the improved selectivity was due to the larger electronegativity of doped Ir, enhancing adsorption of reactants including NO_3_^−^ and H^+^.

**Figure 6:**
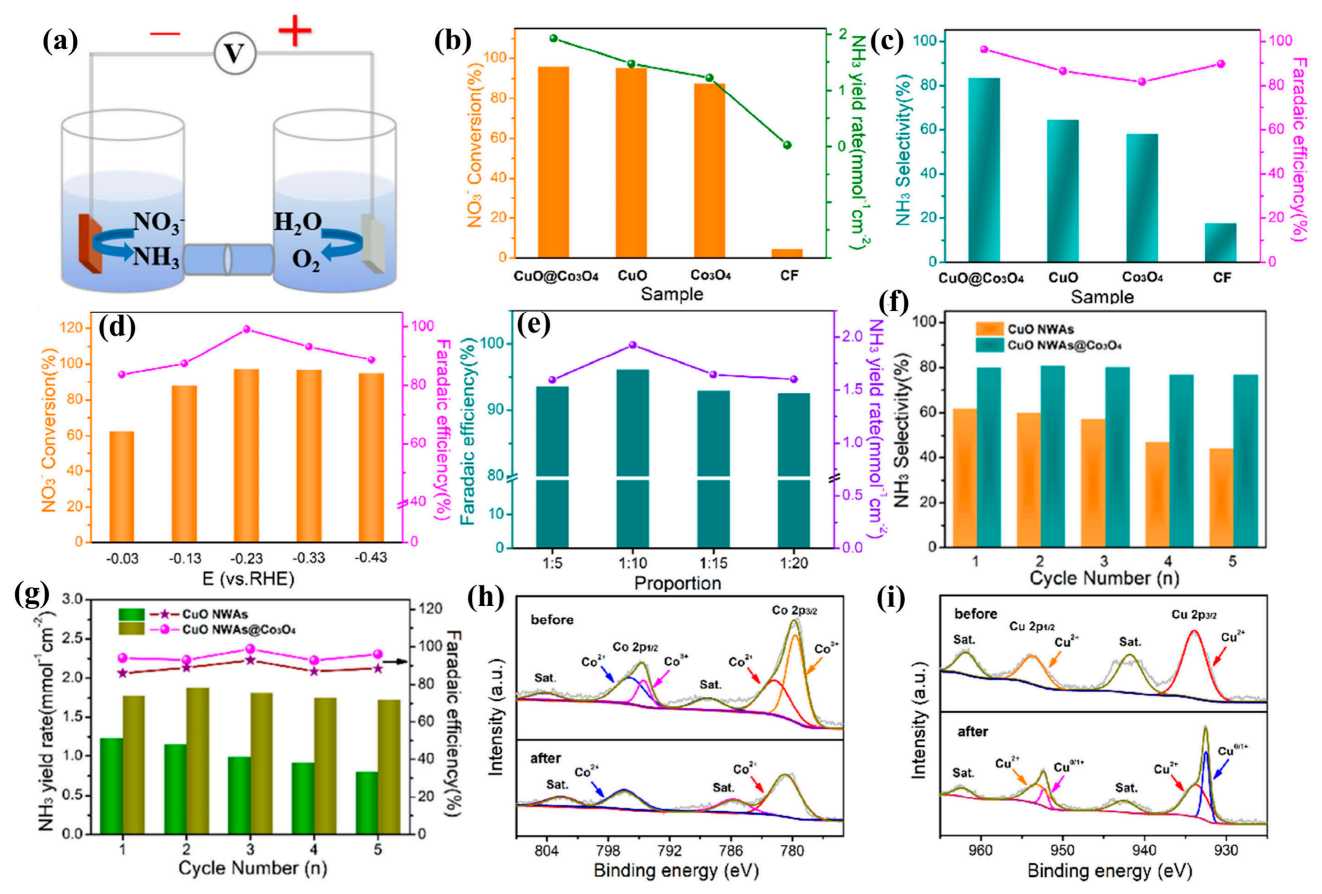
(**a**) Schematic illustration for H-type electrochemical cell. (**b**) Nitrate rate of conversion and ammonia yield rate over different samples. (**c**) Selectivity of ammonia and Faradaic efficiency of ammonia over different samples. (**d**) Potential-dependent Faradaic efficiency of ammonia and conversion rate of nitrate over CuO NWAs@Co_3_O_4_. (**e**) Faraday efficiency and yield rate of ammonia over CuO NWAs@Co_3_O_4_ at −0.23 V vs. RHE with (1:5, 1:10, 1:15 and 1:20) precursor solution proportion. (**f**,**g**) Stability testing over CuO NWAs and CuO NWAs@Co_3_O_4_ cathode at −0.23 V. (**h**) Co 2p and (**i**) Cu 2p spectra of the CuO NWAs@Co_3_O_4_ electrode before and after reaction at −0.23 V for 2.5 h. Adapted from Ref. [125].

Catalysts combining alloys with CuO_x_ have also been extensively studied for eNO_3_^−^RR. For example, the Peng Yue group at Tsinghua University reported PdCu/Cu_2_O with a mesoporous hollow sphere structure for reducing 100 ppm nitrate [121]. At −0.8 V vs. RHE, the selectivity and FE for NH_3_ generation were 96.7% and 94.32%, respectively. This is because Pd can induce Cu’s 3d orbitals to polarize more easily, leading to an increase in active sites, which promotes the formation of *N intermediates and improves NH_3_ selectivity. Won et al. reported that Cu_x_Pd_y_O_x_ with a Cu: Pd ratio of 0.65:0.35 (Cu_0.65_Pd_0.35_O_x_) exhibited a high NH_3_ yield of 1.41 mg h^−1^ cm^−2^ at −0.2 V vs. RHE, which was 14 times that of CuO_x_, because Pd promotes the formation of *NOOH intermediates [122]. The Schuhmann research team reported a Cu_2_O + Co_3_O_4_ tandem catalyst with stable NH_3_ yields, 7.5 times and 2.7 times higher than Cu_2_O and Co_3_O_4_, respectively [123]. To reveal the synergistic effect between CoO_x_ and CuO_x_, they prepared core–shell Cu/CuO_x_ and Co/CoO hybrids by electrochemically reducing CuCo binary sulfides [124]. They found that the inner Cu/CuO_x_ could promote the reduction of NO_3_^−^ to NO_2_^−^, which was then reduced to NH_3_ by the Co/CoO shell, achieving an NH_3_ yield of 1.17 mmol h^−1^ cm^−2^ at −0.165 V vs. RHE. Furthermore, a hybrid of Co_3_O_4_ (core) and CuO nanowire arrays (shell) (CuONWAs@Co_3_O_4_) also delivered an NH_3_ yield rate of 1.915 mmol h^−1^ cm^−2^ at −0.23 V vs. RHE and retained 93.39% nitrate conversion, 76.94% NH_3_ selectivity, and 1.725 mmol h^−1^ cm^−2^ NH_3_ yield after five consecutive cycles (Figure 6a–g). The enhanced nitrate reduction performance originates from the synergistic coupling between CuO nanowire arrays that facilitate nitrate reduction and Co_3_O_4_ domains that promote adsorbed hydrogen (H*) formation (Figure 6h,i) [125].

#### 3.3.2. Titanium Oxides (TiO_x_)

**Figure 7:**
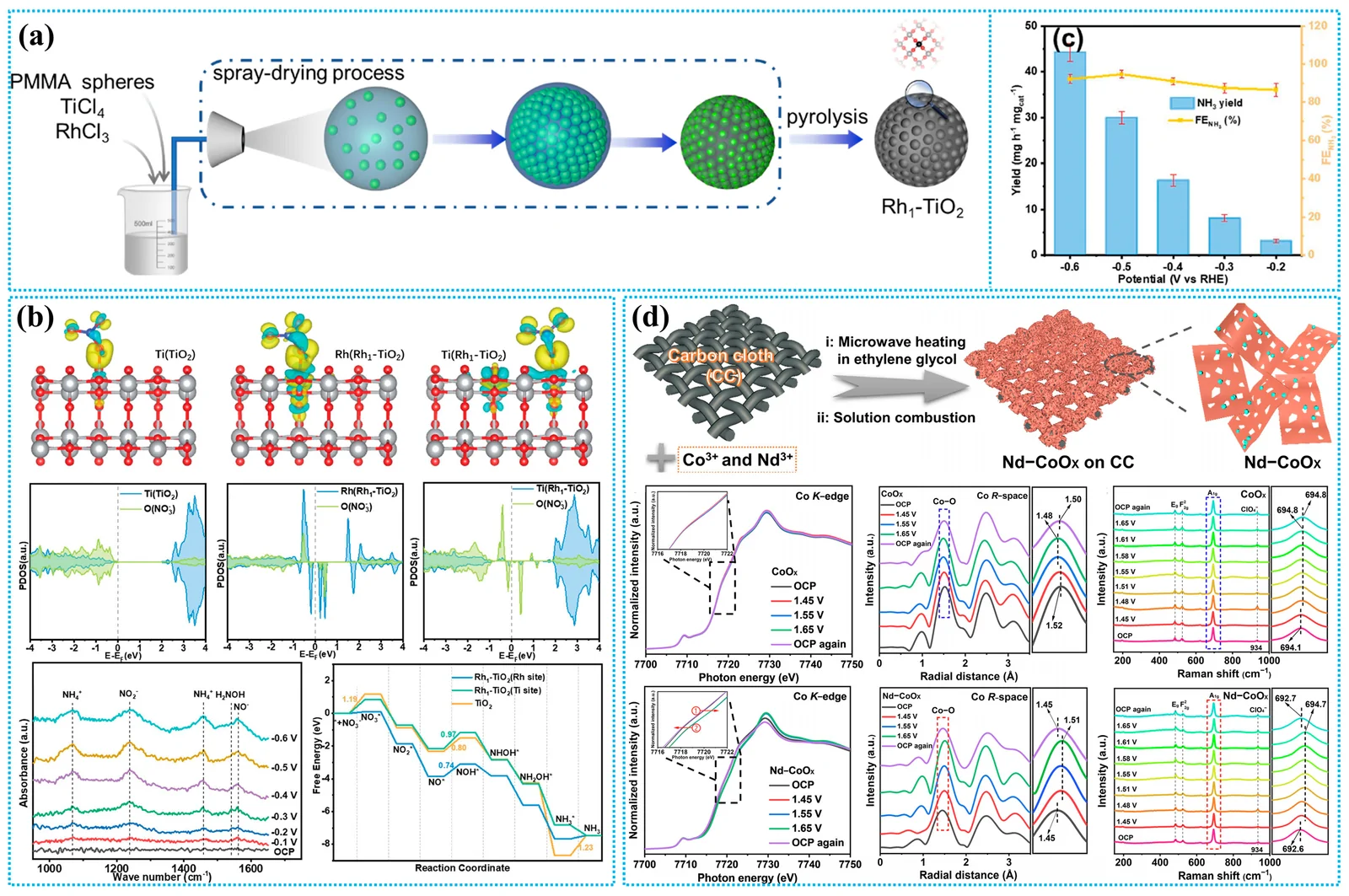
(**a**) Depicts the preparation of Rh_1_-TiO_2_ through spray-drying and pyrolysis process; (**b**) DFT calculations and the working mechanism; (**c**) NH_3_ yield rate and FE of Rh_1_-TiO_2_ electrodes under various potentials during NO_3_RR [127]. (**d**) Schematic of the fabrication process for Nd-CoO_x_ catalysts and in-situ XAS and Raman measurements. Adapted from Ref. [141].

TiO_2_ has been widely studied as an absorbent or support in various photocatalytic reaction systems [126,127,128]. However, TiO_2_-based materials can also serve as efficient catalysts for NH_3_ synthesis through different electrochemical processes, including eNRR [129,130], eNORR [131,132] and eNO_2_^−^RR [133,134]. Although pure TiO_2_ has low conductivity and difficulty adsorbing N_2_, NO, and NO_2_^−^, introducing O vacancies [129,132] and doping with heteroatoms [134] can promote electron transfer and enhance reactant adsorption. Researchers have demonstrated that these strategies are also effective for improving eNO_3_^−^RR. Zhang et al. reported that annealing TiO_2_ in an H_2_ atmosphere could improve its catalytic performance in eNO_3_^−^RR because it created abundant O vacancies that effectively trap NO_3_^−^, leading to weakened N–O bonds [135]. Online differential electrochemical mass spectrometry (DEMS) and DFT calculations further indicated that NO_3_^−^ occupies the O vacancies on TiO_2−x_, causing N–O bond weakening, suppressing byproduct formation, and enhancing catalyst activity and NH_3_ yield. Zhi et al. developed Pd-doped TiO_2_ nanoarrays for electrocatalytic nitrate reduction, achieving an NH_3_ yield of 1.12 mg cm^−2^ h^−1^ and a FE of 92.1% in a 0.25 M NO_3_^−^ electrolyte. DFT calculations revealed that Pd doping optimized the adsorption energies of key eNO_3_^−^RR intermediates (e.g., *NO_3_, NO_2_, and NO), thereby facilitating intermediate desorption and promoting nitrate reduction kinetics [136]. Zhao et al. developed atomically dispersed Rh sites on porous TiO_2_, achieving a high NH_3_ Faradaic efficiency of 94.7% and a yield rate of 29.98 mg h^−1^ mg^−1^ at −0.5 V vs. RHE (Figure 7a,b). Mechanistically, isolated Rh atoms enhance NO_3_^−^ adsorption and charge transfer while activating adjacent Ti sites, thereby lowering the rate-limiting energy barrier and promoting selective nitrate-to-ammonia conversion (Figure 7c) [127]. Trace Cu doping (0.02%) can not only improve the conductivity of TiO_2_ but also promote the generation of O vacancies due to charge differences [137]. After 12 h of electrolysis, the Cu-doped TiO_2_ catalyst significantly increased the conversion rate of nitrate to NH_3_ and exhibited high NH_3_ selectivity, attributed to increased active surface area and lowered energy barriers.

Due to its reducibility and tunable catalytic properties, TiO_2_ has also shown promise as a support and cocatalyst for eNO_3_^−^RR in nitrate removal. Sun et al. anchored Co nanoparticles on TiO_2_ nanobelt arrays, achieving an NH_3_ yield as high as 800 μmol h^−1^ cm^−2^ and an FE of 96.7% at −1 V vs. RHE [138]. The Schottky contact between metallic Co and semiconducting TiO_2_ generated a built-in electric field, accelerating electron delocalization and yielding high NH_3_ production. Cu clusters supported on TiO_2−x_ nanosheets with abundant O vacancies showed an FE of 81.34% at −0.95 V vs. RHE [139]. DEMS and DFT calculations indicated that O vacancies at the Cu/TiO_2−x_ interface enhance NO_3_^−^ adsorption and dissociation, thus creating different energy pathways and lowering the limiting potential. Liu et al. proposed a sequential active site switching mechanism based on TiO_2_-anchored Cu/Fe hetero-nanoparticles [140]. Their study showed that NO_3_^−^ tends to adsorb on the in-plane Fe phase and reduce to NO_2_^−^, which then transfers to the Cu/Fe interface for subsequent reduction to *NH_3_, finally desorbing from the in-plane Cu phase. This mechanism makes the reduction of NO_3_^−^ to NH_3_ easier, achieving an NH_3_ yield of 505.73 μg h^−1^ cm^−2^ and an FE of 91.2% at −1.4 V vs. SCE.

#### 3.3.3. Cobalt Oxides (CoO_x_)

CoO_x_ has attracted considerable attention in the eNO_3_^−^RR field due to its excellent NO adsorption capacity, high conductivity, tunable electronic structure, low cost, and good stability [141,142,143]. Among them, spinel-structured cobalt oxide (Co_3_O_4_), composed of CoO_6_ octahedra and CoO_4_ tetrahedra [144] has been extensively studied for the structure–activity relationship between its unique crystal structure and catalytic performance. Zhou et al. revealed the catalytic mechanism of Co_3_O_4_: Co^3+^ sites first adsorb NO_3_^−^, which is then reduced to NH_3_ via the adsorbed hydrogen mechanism [141]. This mechanism explains why Co_3_O_4_ exhibits higher NH_3_ yields compared to CoO and Co_2_O_3_. Studies show that optimizing the Co^2+^/Co^3+^ ratio can effectively enhance catalytic performance. Li et al. found that Co_3_O_4_ nanorods with a higher Co^2+^/Co^3+^ ratio (mainly exposing the (220) facet) exhibited better catalytic activity than Co_3_O_4_ nanoparticles and nanosheets [142].

O vacancy engineering is another effective strategy to enhance CoO_x_ catalytic performance. Studies show that surface O vacancies can significantly inhibit HER, thereby increasing eNO_3_^−^RR activity [145]. Feng et al. combined oxygen-vacancy engineering with Nd insertion to regulate the local Co–O coordination environment, enabling reversible lattice “breathing” that stabilizes defect-rich CoO_x_ during acidic oxygen evolution (Figure 7d) [141]. Shao et al. prepared CoO_x_ nanosheets rich in O vacancies, achieving an FE of 93.4% at −0.2 V vs. RHE [145]. Mechanistic studies indicated that surface O vacancies not only suppress H_2_ evolution but also promote the protonation of *NO_3_^−^ and *NO, significantly increasing NH_3_ yield. Furthermore, heterostructure design offers new avenues for performance optimization. Co/CoO nanosheet arrays exhibited excellent catalytic performance at −1.3 V vs. RHE, with an NH_3_ yield of 194.46 μmol h^−1^ cm^−2^, an FE of 93.8%, and NH_3_ selectivity of 91.2%. This performance enhancement is mainly attributed to the electron deficiency induced by the Schottky contact, which not only suppresses competing HER and byproduct formation but also promotes NH_3_ generation [143].

To optimize the catalytic performance of Co_3_O_4_ in eNO_3_^−^RR, researchers have proposed various strategies, among which defect engineering has garnered significant attention due to its remarkable effect on enhancing catalytic performance [146,147,148,149,150]. Sun et al. found that Co_3_O_4_ nanosheets with Co vacancies exhibited an NH_3_ yield of 517.5 μmol h^−1^ cm^−2^ and an FE of 97.2% at −0.6 V vs. RHE, significantly outperforming unmodified Co_3_O_4_ nanosheets (NH_3_ yield: 183.8 μmol h^−1^ cm^−2^ and FE: 85.9%) [148]. The Chen Yiwen group at Harbin Institute of Technology prepared Co_3_O_4_ electrocatalysts rich in O vacancies (57%) and with a high Co^2+^/Co^3+^ ratio using He plasma treatment [147]. This catalyst demonstrated excellent performance in a 100 ppm NO_3_^−^ solution, achieving over 99% conversion within 3 h, significantly outperforming samples without O vacancies. Further studies indicated that O vacancy-modified Co_3_O_4_ shows promise for electrocatalytic nitrate reduction to NH_3_. Sun et al. reported that O vacancy-modified Co_3_O_4_ achieved an NH_3_ yield of 13,229 μg h^−1^ cm^−2^ at −0.6 V vs. RHE, demonstrating potential for nitrate-containing wastewater treatment [150]. Wang et al. induced multi-scale O vacancies in Co_3_O_4_ nanosheets by controlling the calcination atmosphere and systematically investigated the influence of O vacancy density on electrocatalytic performance [151]. Experimental results showed that Co_3_O_4_ nanosheets with the highest O vacancy content exhibited the best catalytic performance at −0.9 V vs. RHE, with an NH_3_ yield of 4.43 mg h^−1^ cm^−2^ and an FE of 88.7%. This performance enhancement was mainly attributed to the abundant O vacancies effectively suppressing the HER process while lowering the energy barrier for the rate-determining step “*NO to *NHO”.

To enhance the performance of metal oxides in eNO_3_^−^RR, researchers commonly employ element doping strategies to tune the electronic structure of catalysts, thereby optimizing the adsorption behavior of reactants and reaction intermediates and lowering reaction energy barriers. The spinel Co_3_O_4_ crystal structure consists of CoO_4_ tetrahedra and CoO_6_ octahedra, allowing dopant elements to occupy different lattice sites. Studies show that partial substitution of Co^2+^ sites within the CoO_4_ tetrahedra can effectively improve NO_3_^−^ conversion. Sun et al. found that Fe doping significantly increased the NH_3_ production rate of Co_3_O_4_ [152]. X-ray absorption spectroscopy confirmed that Fe preferentially substitutes surface Co atoms in the CoO_4_ tetrahedra, a structural modulation that not only enhances NO_3_^−^ adsorption but also effectively suppresses HER. Similarly, Cu-doped Co_3_O_4_ systems, where Cu occupies metal sites in the CoO_4_ tetrahedra, form a lower d-band center, significantly shifting the limiting potential of eNO_3_^−^RR negatively while lowering the Gibbs free energy for HER, ultimately achieving an FE of 86.5% and high NH_3_ yield [153]. It is noteworthy that since many transition metals lack suitable doping sites in the CoO_6_ octahedra, traditional research has focused less on modifying these sites [148,154]. However, DFT calculations indicate that increasing the Co^2+^/Co^3+^ ratio can significantly promote NH_3_ synthesis efficiency. Given that CoO_4_ tetrahedra and CoO_6_ octahedra correspond to the coordination environments of Co^2+^ and Co^3+^, respectively, substituting Co^3+^ in the CoO_6_ octahedra can effectively modulate the Co^2+^/Co^3+^ ratio [155]. Recent work by the Pan Hui group at the University of Macau [156] validated this strategy: in a neutral electrolyte, Mn-doped Co_3_O_4_ achieved an NH_3_ yield of 35 mg h^−1^ cm^−2^ and an FE as high as 99.5% at −1.2 V vs. RHE, significantly outperforming pure Co_3_O_4_. Raman spectroscopy analysis revealed that Mn selectively substitutes some Co atoms in the CoO_6_ octahedral sites, and this specific doping-induced electronic structure reconstruction is the key mechanism for enhanced eNO_3_^−^RR activity. Furthermore, the catalyst exhibited excellent stability in continuous testing, laying the foundation for its practical application.

#### 3.3.4. Bimetallic Spinel Oxides

Based on the modification strategies of the Co_3_O_4_ lattice structure, researchers have successfully prepared MCo_2_O_4_ and CoM_2_O_4_ spinel catalysts by fully substituting Co^3+^ ions in the CoO_4_ tetrahedra or CoO_6_ octahedra. Experiments show that CuCo_2_O_4_ catalysts supported on carbon nanofibers exhibit significant catalytic activity in eNO_3_^−^RR, attributed to enhanced electron transfer between Co and N atoms [155]. Sun et al. systematically constructed a series of MCo_2_O_4_ spinel systems (M = Ni, Fe, Zn) supported on carbon cloth [157,158,159]. Compared to pristine Co_3_O_4_, the FE of all catalysts in this series increased to above 95%. Among them, NiCo_2_O_4_ exhibited the highest NH_3_ yield (17.52 mg h^−1^ cm^−2^) at −0.6 V vs. RHE, which is closely related to its enhanced conductivity and reduced limiting potential for NH_3_ generation. Notably, the CoMn_2_O_4_ catalyst confined in multi-channel carbon nanofibers achieved an NH_3_ yield of 1041.1 μg h^−1^ cm^−2^ and an FE of 92.4% at −0.7 V vs. RHE, with the performance enhancement mechanism attributed to substrate–catalyst interface-induced electron delocalization, which effectively suppresses HER and optimizes the energy barrier for the eNO_3_^−^RR rate-determining step [160].

**Figure 8:**
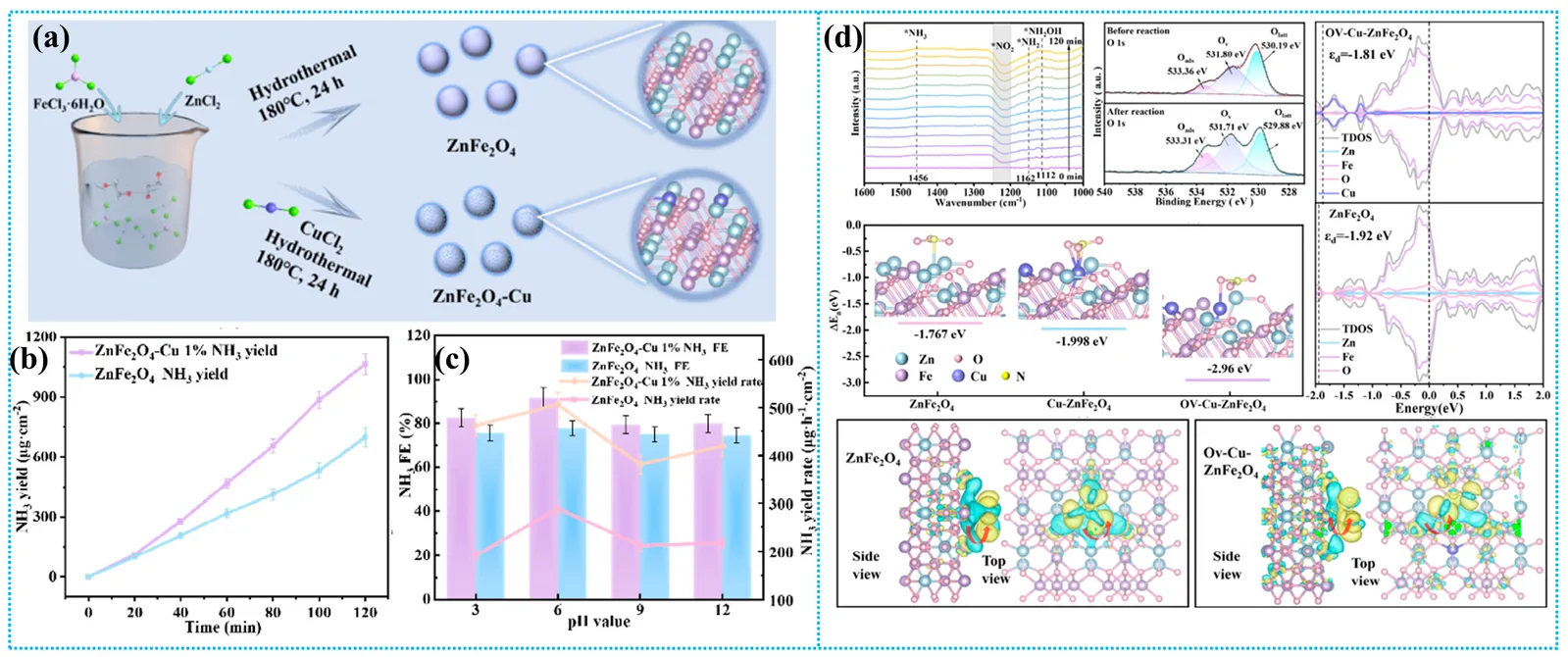
(**a**) The preparation process of the ZnFe_2_O_4_ and ZnFe_2_O_4_-Cu x%; (**b**) NH_3_ yield rate and FE of ZnFe_2_O_4_ and ZnFe_2_O_4_-Cu 1% in different initial electrolyte pH values; (**c**) Curves of NH3 yields over time for ZnFe_2_O_4_-Cu 1% and ZnFe_2_O_4_ under optimal reaction conditions; (**d**) Mechanistic analysis of electrocatalytic NO3RR Adapted from Ref. [162].

In addition to Co-based systems, Fe_3_O_4_ and its derivative NiFe_2_O_4_ as typical spinel oxides have also attracted considerable attention in the eNO_3_^−^RR field [161]. Zhou et al. developed a Cu-doped ZnFe_2_O_4_ catalyst in which Cu incorporation simultaneously induces oxygen vacancies and modulates the electronic structure, creating complementary active sites for nitrate adsorption and activation (Figure 8a). The oxygen vacancies enhance NO_3_^−^ capture, while Cu sites promote electron transfer and N–O bond cleavage, thereby facilitating the nitrate-to-ammonia pathway (Figure 8d). The optimized ZnFe_2_O_4_–Cu catalyst achieved an NH_3_ yield rate of 531.78 μg h^−1^ cm^−2^ and a maximum Faradaic efficiency of 91.52%, outperforming pristine ZnFe_2_O_4_ (Figure 8b,c) [162]. Shao et al. reported that Cu doping (6.1 wt%) significantly enhanced the Faradaic efficiency of Fe_3_O_4_ to 100%, which was attributed to enhanced NO_3_^−^ adsorption and a reduced energy barrier for the rate-determining step [163]. It is particularly noteworthy that the Fe_3_O_4_@TiO_2_ heterostructure exhibited significant advantages under neutral conditions: at −0.9 V vs. RHE, its NH_3_ yield was as high as 12.394 mg h^−1^ cm^−2^, with an FE of 88.4%, far exceeding the performance of either Fe_3_O_4_ or TiO_2_ alone [164].

Further studies integrating in situ characterization and theoretical calculations are needed to deeply analyze the structure–activity relationships and dynamic catalytic mechanisms of spinel oxides, thereby guiding the rational design of high-performance catalysts. Concurrently, developing stable catalytic systems capable of industrial-level current densities is a key research direction for promoting the practical application of eNO_3_^−^RR technology.

#### 3.3.5. Perovskite Oxides

Perovskite-type oxides (ABO_3_) have shown broad application prospects in the field of electrocatalysis due to their low cost, excellent chemical stability, and environmental friendliness [110,165]. However, pristine perovskite oxides exhibit weak adsorption of NO_3_^−^ at metal–oxygen surface sites, significantly limiting their catalytic performance in eNO_3_^−^RR to NH_3_.

Studies have shown that defect engineering, such as introducing O vacancies, can effectively enhance the eNO_3_^−^RR activity of perovskite-based catalysts [166]. The Liu Tianxi group at Jiangnan University systematically investigated the catalytic performance of La-based perovskite oxides (LaCrO_3_, LaMnO_3_, LaFeO_3_, LaCoO_3_) and found that the activity followed the order LaCrO_3_ < LaMnO_3_ < LaFeO_3_ < LaCoO_3_ [167]. Notably, the catalytic activity was positively correlated with the concentration of O vacancies in the material, which significantly promotes the rate-determining step “*HNO_3_ to *NO_2_”. At −1.1 V vs. RHE, a current density of 150 mA cm^−2^ and an FE of 90% were achieved. The Zhou Hao group increased the O vacancy density by modulating the La defect concentration (10%) in LaFeO_3_, which not only enhanced eNO_3_^−^RR activity but also suppressed the competing HER [168]. This phenomenon has also been verified in other perovskite systems [166,169].

B-site doping is another effective strategy to optimize the eNO_3_^−^RR performance of perovskite oxides. Chen et al. found that introducing dopants such as Co, Ni, and Zn into the layered perovskite LaCu_2_O_4_, with the Co-doped sample exhibiting the best NH_3_ partial current density and FE [170]. Additionally, structural reconstruction strategies have been shown to significantly enhance the catalytic activity of perovskite oxides [169,171]. For example, crystalline BiFeO_3_ flakes spontaneously reconstruct into an amorphous phase during electrocatalysis, achieving an NH_3_ yield of 132.3 mg h^−1^ mg^−1^ and an FE of 96.85% at −0.6 V vs. RHE [171].

#### 3.3.6. Other Metal Oxides

Transition metal oxides also show great potential in the eNO_3_^−^RR field. The Song Wenbo group designed and developed two Mo-based electrocatalysts: MoO_2_-C nanoball flowers (MoO_2_-C NBF) dominated by Mo(IV) sites and MoO_3_-C nanoball flowers (MoO_3_-C NBF) dominated by Mo(VI) sites [172]. Theoretical calculations and experimental results indicated that Mo(IV) sites exhibit significantly better catalytic activity and selectivity for NH_3_ than Mo(VI) sites. MoO_2_-C NBF achieved an NH_3_ yield of 109.28 μmol h^−1^ cm^−2^ and an FE of 99.05% at −0.3 V vs. RHE, attributed to the Mo(IV) sites effectively inhibiting NO_2_^−^ desorption and promoting *NO protonation. Chu et al. reported MoO_2_ nanosheets rich in O vacancies, whose NH_3_ yield was 10 times higher than that of pristine MoO_2_, with a FE of 92.4%, mainly due to the enhanced nitrate adsorption by O vacancies [173]. Furthermore, hybridization of MoO_2_ with metals such as Cu and Pd has been studied to improve the catalytic performance of eNO_3_^−^RR [174,175].

**Figure 9:**
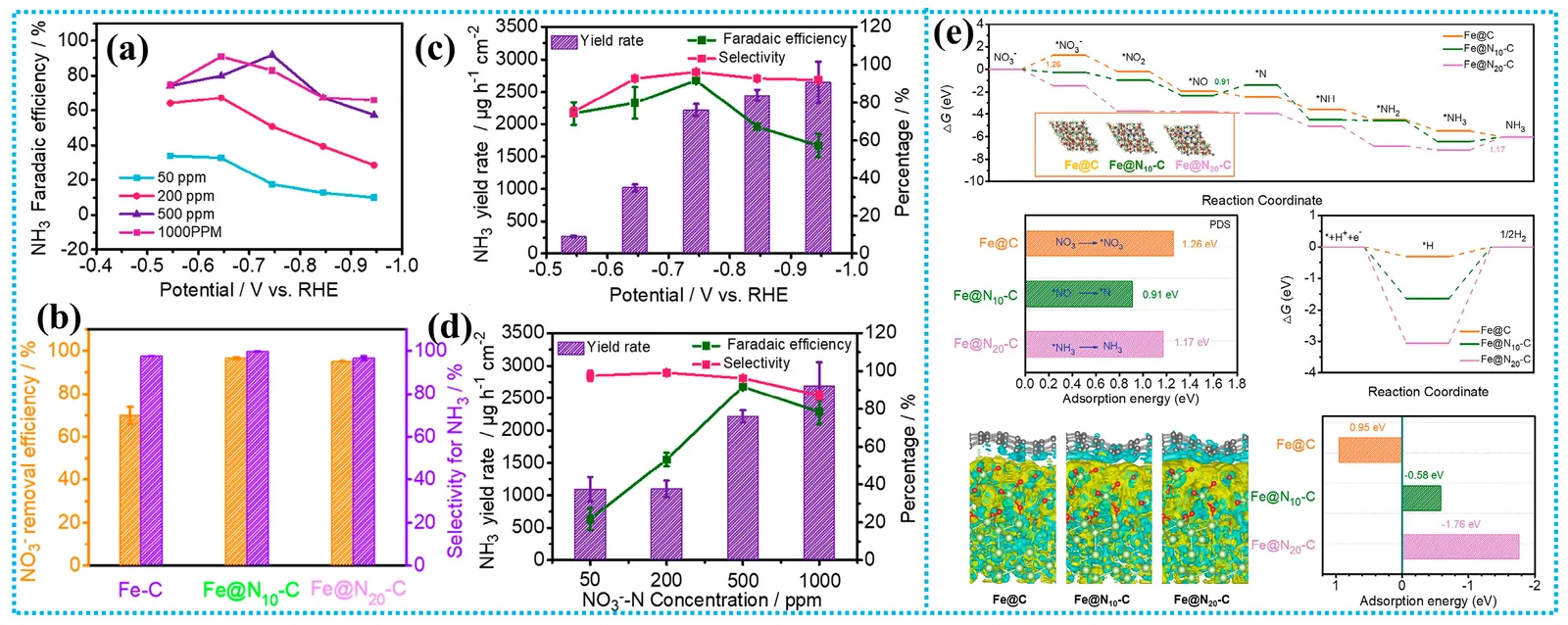
(**a**) NH_3_ FE with different NO_3_^−^ concentrations at different applied potentials. (**b**) NO_3_^−^ removal efficiencies and NH_3_ selectivities. (**c**) Potential-dependent FE, NH_3_ yield rate, and NH_3_ selectivity. (**d**) Comparison of the highest FE, yield rate, and NH_3_ selectivity at different NO_3_^−^ concentrations. (**e**) Mechanistic study of catalytically active sites in Fe@N_10_-C for NO_3_^−^RR [176].

Heterostructure design can further enhance the catalytic performance of transition metal oxides. Studies show that Cu/MnO_x_ nanoparticles exhibit an NH_3_ yield of 5.53 mg h^−1^ mg^−1^ and an FE of 98.2% at −0.6 V vs. RHE [176]. The excellent performance arises from the np-Cu/MnO_x_ interface, accelerating electron transfer, suppressing HER, and promoting *NO_2_ protonation. Zhang et al. constructed Fe nanoparticles encapsulated by optimally N-doped carbon (Fe@N_10_-C), achieving an NH_3_ yield rate of 2647.7 μg h^−1^ cm^−2^, a Faradaic efficiency of 91.8%, and up to 99.7% NH_3_ selectivity (Figure 9a–d). Mechanistically, N doping activates adjacent carbon sites and strengthens Fe-to-carbon interfacial charge transfer, thereby optimizing NO_3_^−^ adsorption while suppressing the competing hydrogen evolution reaction (Figure 9e) [177]. Li et al. prepared Fe_2_TiO_5_ nanofibers rich in O vacancies, achieving an NH_3_ yield of 0.73 mmol h^−1^ mg^−1^ and an FE of 87.6% at −0.9 V vs. RHE, with surface Fe atoms forming anti-bonding states that significantly accelerate charge transfer [178]. These results fully confirm the great potential of transition metal oxides in electrocatalytic nitrate reduction to NH_3_ (Table 2).

### 3.4. Metal Phosphide Catalysts

**Figure 10:**
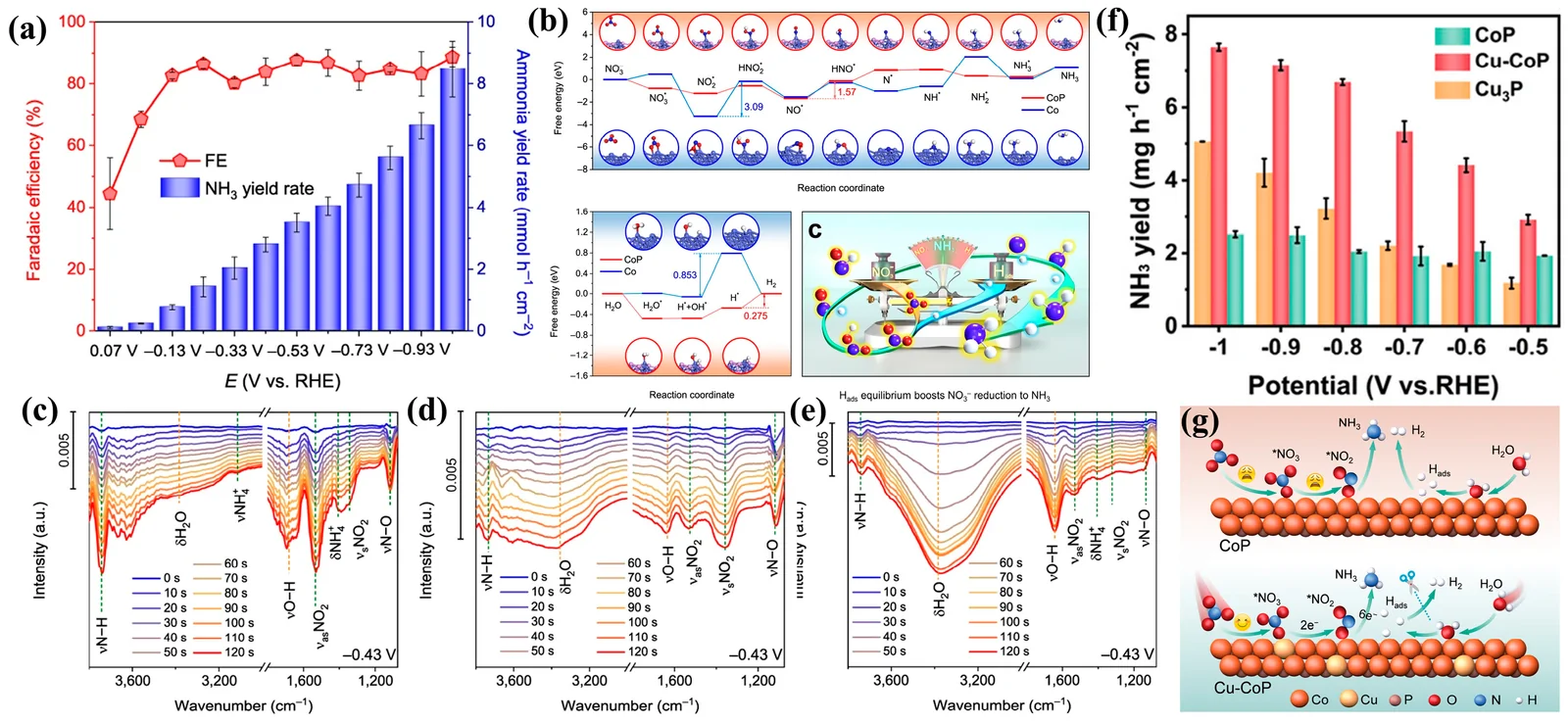
(**a**) NH_3_ FEs and corresponding yield rates of CoP-CNS on Cu foam under potential range from 0.07 to −1.03 V vs. RHE estimated by three independent tests; (**b**) DFT calculations and (**c**–**e**) in situ FTIR spectra collected in different configurations for different catalysts Adapted from Ref. [185]. (**f**) NH_3_ yield at different applied potentials in 0.1 M K_2_SO_4_ + 0.01 M KNO_3_. (**g**) Schematic illustration of the NO_3_^−^RR process over the CoP and Cu-CoP catalyst systems. Adapted from Ref. [190].

Metal phosphides have shown significant advantages in various catalytic reactions due to their excellent conductivity and Pt-like electronic structure characteristics [180,181]. The performance of cobalt phosphide (CoP) in eNO_3_^−^RR has attracted extensive attention from researchers [182,183]. The Li Shuni group at Shaanxi Normal University found that CoP nanorings exhibit higher NH_3_ yields than CoP nanoparticles, mainly due to their larger active surface area and better NH_3_ selectivity [182]. Further studies showed that amorphous CoP nanospindles achieved an NH_3_ yield of 19.28 mg h^−1^ mg^−1^ and an FE of 94.24% at −0.5 V vs. RHE, with excellent stability for up to 20 hours [184]. Shao et al. developed a CoP nanosphere catalyst supported on carbon nanosheet arrays (CoP-CNS), achieving an NH_3_ yield of 8.47 mmol h^−1^ cm^−2^ and an FE of 88.6% at −1.03 V vs. RHE (Figure 10a). In-situ experimental characterization and theoretical calculations confirmed that the abundant H atoms on the CoP surface facilitate the electrochemical reduction of nitrate to NH_3_ (Figure 10b–e) [185].

Besides CoP, Fe-based [186] and Ni-based [48,187,188] phosphides also exhibit excellent performance in eNO_3_^−^RR. Studies show that both FeP and Fe_2_P films can efficiently and stably catalyze the reduction of nitrate to NH_3_, with Fe_2_P achieving an NH_3_ yield of 2.10 mg h^−1^ cm^−2^ and an FE of 96% at −0.55 V vs. RHE [186]. The performance advantage stems from easier *NO protonation and faster *NH_3_ desorption kinetics. Zhou et al. prepared a self-supported Ni_2_P catalyst with a (111) facet, achieving an FE as high as 99.23%, attributed to the (111) facet effectively promoting NO_3_^−^ adsorption and *H formation, thus generating NH_3_ efficiently via the adsorbed hydrogen mechanism [48]. To further enhance the catalytic performance of metal phosphides, researchers have developed metal doping strategies. The Zhi Chunyi group found that Fe-doped Ni_2_P achieved an NH_3_ yield of 212.3 μg h^−1^ cm^−2^ and an FE of 94.3% at −0.4 V vs. RHE. This was mainly attributed to the upshift of the d-band center, reducing the adsorption energy of intermediates, thereby lowering the energy barrier for electron transfer from Ni to Fe [189]. Cu–Co dual-site engineered CoP catalysts achieved efficient nitrate electroreduction to ammonia under neutral low-concentration conditions, delivering a high NH_3_ yield rate of 7.65 mg h^−1^ cm^−2^ and an FE of 85.1% (Figure 10f). The enhanced activity originates from the tandem catalytic mechanism, in which Cu sites facilitate NO_3_^−^ adsorption/activation and Co sites accelerate NO_3_^−^ hydrogenation, while Cu-induced electronic modulation optimizes H* generation and suppresses HER (Figure 10g) [190].

Furthermore, significant progress has been made in the study of composite phosphides. Sun et al. constructed a CoP/TiO_2_ nanoarray p–n heterojunction on a Ti plate (CoP/TiO_2_@TP), significantly enhancing the activity of CoP in eNO_3_^−^RR. At −0.5 V vs. RHE, this catalyst achieved an NH_3_ yield of 499.8 μg h^−1^ cm^−2^ and an FE of 95%, significantly outperforming pure CoP and TiO_2_ [191]. The performance enhancement mechanism is mainly attributed to the built-in electric field induced by the p–n heterojunction due to the Fermi level difference, which effectively promotes electron transfer.

### 3.5. Metal Sulfide Catalysts

Significant progress has been made in the study of the catalytic performance of metal sulfides in electrocatalytic nitrate reduction to NH_3_. Comparative studies by the Cheng Daojian group and the Zhu Weihua group revealed that 1T-phase B@MoS_2_ exhibits superior catalytic performance in eNO_3_^−^RR compared to 2H-phase B@MoS_2_. This is mainly attributed to its narrower bandgap and lower limiting potential, a conclusion supported by DFT calculations [192]. Chu et al. developed a Ce-doped MoS_2−x_ nanoflower array catalyst rich in S vacancies, achieving an NH_3_ yield of 7.3 mg h^−1^ cm^−2^ and an FE of 96.6% at −0.7 V vs. RHE [193]. Theoretical calculations indicated that S vacancies effectively activate NO_3_^−^ adsorbed on Mo active sites, while Ce doping significantly enhances the adsorption rate of *NO, thereby accelerating the protonation process. The same group further reported B-doped MoS_2_ achieving an NH_3_ yield of 10.8 mg h^−1^ cm^−2^ and an FE of 92.3% at the same potential [194]. The performance enhancement is mainly attributed to effective suppression of HER and promotion of eNO_3_^−^RR.

Heterostructure design offers a new approach to enhance the catalytic performance of metal sulfides. Studies show that the Bi_2_S_3_/MoS_2_ heterostructure achieves an NH_3_ yield of 0.15 mmol h^−1^ cm^−2^ and an FE of 88.4% at −0.8 V vs. RHE, significantly outperforming pure Bi_2_S_3_ and MoS_2_ [195]. This is mainly due to charge transfer from Bi_2_S_3_ to MoS_2_, lowering the limiting potential of the reduction pathway. Furthermore, La-doped VS_2−X_ nanosheets rich in S vacancies exhibited an NH_3_ yield of 11.3 mg h^−1^ cm^−2^ and an FE of 96.6% at −0.6 V vs. RHE, surpassing pristine VS_2_ [196]. This is attributed to the synergistic effect of La doping and S vacancies: on one hand, HER is suppressed; on the other hand, NO_3_^−^ adsorption is enhanced and the limiting potential for NH_3_ generation is lowered. The Sun Xuping research group developed a FeS_2_ nanoparticle catalyst supported on TiO_2_ nanobelts (FeS_2_@TiO_2_/TP), achieving an NH_3_ yield of 860.3 μmol h^−1^ cm^−2^ at −0.7 V vs. RHE. The excellent performance stems from promoted charge transfer and a lowered energy barrier for *NO protonation [197].

**Figure 11:**
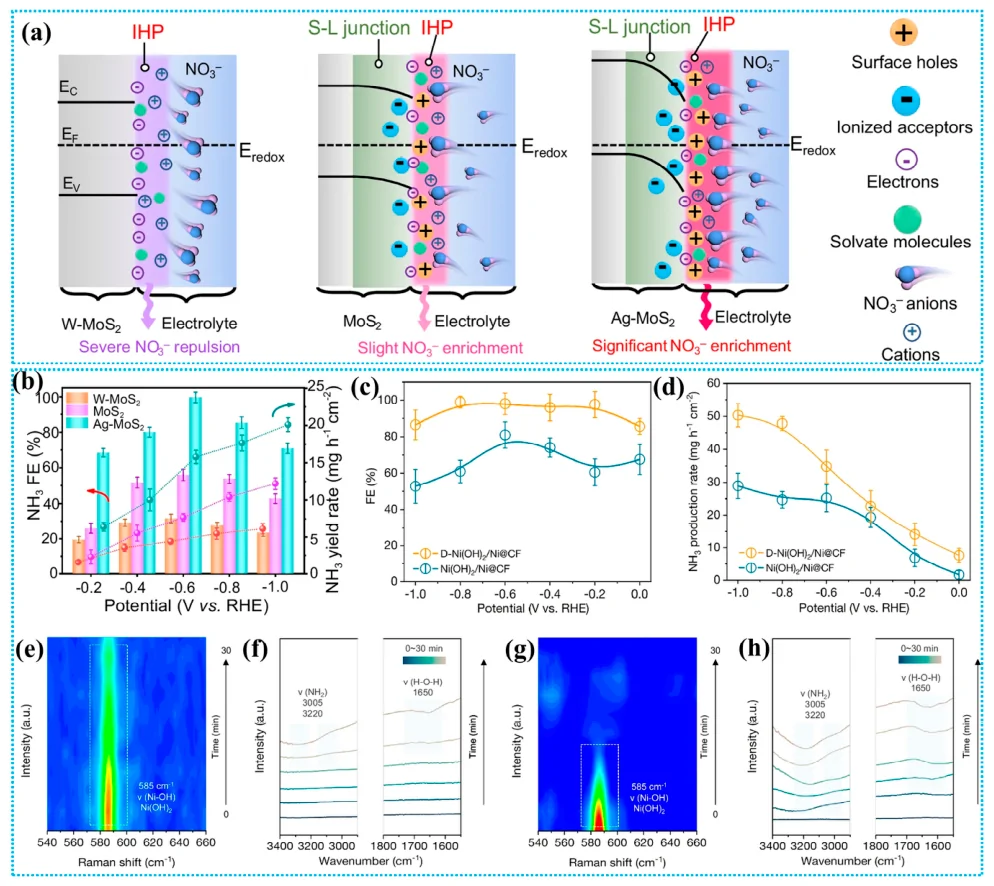
(**a**) NO_3_^−^ distribution on IHP region over MoS_2_-based catalysts; (**b**) NH_3_ yield rate and NH_3_ FE of catalysts in 10 mM NO_3_^−^ electrolyte at various applied potentials Adapted from Ref. [198]. (**c**) FE and (**d**) NH_3_ production rate on Ni(OH)_2_/Ni@CF and D-Ni(OH)_2_/Ni@CF in 1 M KOH + 0.1 M KNO_3_ electrolyte at the potential range from 0.0 V vs. RHE to −1.0 V vs. RHE; In-situ electrochemical Raman spectra at −0.8 V vs. RHE (without *iR* compensation) of (**e**) Ni(OH)_2_/Ni@CF and (**g**) D-Ni(OH)_2_/Ni@CF with a time scale of 0~30 min; In-situ electrochemical FTIR spectra at −0.8 V vs. RHE (without *iR* compensation) of (**f**) Ni(OH)_2_/Ni@CF and (**h**) D-Ni(OH)_2_/Ni@CF with a time scale of ~30 min. Adapted from Ref. [209].

Beyond conventional doping and heterostructure construction, recent studies have increasingly focused on regulating the interfacial microenvironment, crystallographic surfaces, phase structures, and reactor-level mass transport of metal sulfide catalysts. Liao et al. developed Ag-doped MoS_2_, in which a solid–liquid junction induced interfacial charge redistribution and enriched NO_3_^−^ within the inner Helmholtz plane by approximately 28.6-fold (Figure 11a). Consequently, Ag–MoS_2_ achieved a near-unity NH_3_ Faradaic efficiency and an NH_3_ yield rate of approximately 20 mg h^−1^ cm^−2^ under ultralow nitrate concentrations, demonstrating that interfacial reactant enrichment can effectively overcome the mass-transfer limitation of dilute nitrate streams (Figure 11b) [198]. At the device level, monodispersed Bi-doped FeS_2_ integrated into a flow electrolyzer delivered an NH_3_ yield rate of 83.7 mg h^−1^ cm^−2^ with a near-100% Faradaic efficiency at an ampere-level current density of 1023.2 mA cm^−2^, while maintaining stable operation for 100 h, highlighting the potential of metal sulfides for high-throughput ammonia production [199]. In addition, octahedral CoS_2_ exhibited an NH_3_ yield rate of 10.6 ± 0.4 mg h^−1^ cm^−2^ and an FE of 96 ± 1.5%, because the electron-rich CoS_2_(111) surface promoted the further reduction of NO_2_^−^ and inhibited its accumulation [200]. Atomic and phase-level regulation also provides effective routes for optimizing the intrinsic activity of sulfide catalysts. Fe doping in MoS_2_ nanosheets modulated the local electronic structure and improved nitrate-to-ammonia conversion, affording an NH_3_ yield rate of 9.75 mg h^−1^ cm^−2^ and an FE of 90%, whereas theoretical calculations on single-B-decorated MoS_2_ revealed that the metallic 1T phase possessed a lower limiting potential than the semiconducting 2H phase because of favorable electron redistribution and steric regulation [201]. Collectively, these studies suggest that integrating active-site regulation with interfacial nitrate enrichment, facet/phase engineering, and flow-cell design is crucial for advancing metal sulfide catalysts from laboratory-scale activity evaluation toward practical nitrate remediation and continuous ammonia recovery.

### 3.6. Metal Nitride, Hydroxide, and Oxyhydroxide Catalysts

Although metal nitrides have been extensively studied in various electrocatalytic reactions, their application in eNO_3_^−^RR to NH_3_ remains relatively limited [202,203]. Wang et al. reported that N-doped carbon-supported ultrafine metal nitrides achieved a high NH_3_ yield of 9.185 mmol h^−1^ mg^−1^ and an FE of 89.5% at −1.45 V vs. SCE [204]. It is noteworthy that metal nitrides undergo surface reconstruction during water splitting, forming metal hydroxides and oxyhydroxides [202,204,205,206,207], a property that also makes them suitable for electrochemical nitrate reduction to NH_3_ [208,209]. For example, Lv et al. employed Ni(OH)_2_ as a model catalyst and revealed its potential-induced reconstruction into a dynamic Ni(OH)_2_/Ni interface during nitrate electroreduction. By introducing surface hydroxyl vacancies through plasma activation, the resulting D-Ni(OH)_2_/Ni catalyst achieved an NH_3_ Faradaic efficiency of 98.99% and an NH_3_ yield rate of 47.85 mg h^−1^ cm^−2^ at −0.8 V vs. RHE (Figure 11c,d). In-situ spectroscopy, isotope-labeling experiments, and theoretical calculations demonstrated that the activated interface promoted an interfacial OH cycle involving dehydroxylation, water dissociation, and OH adsorption, thereby accelerating proton transfer and the hydrogenation of nitrogen-containing intermediates while suppressing the competing HER (Figure 11e–h) [209]. Oxyhydroxides have also garnered significant attention for their catalytic behavior in eNO_3_^−^RR. Sun et al. found that FeOOH nanorods with intrinsic O vacancies exhibited an NH_3_ yield of 2419 μg h^−1^ cm^−2^ and an FE of 92% in a neutral environment at −0.5 V vs. RHE [210]. The excellent performance originated from the intrinsic O vacancies promoting NO_3_^−^ adsorption and catalyzing the conversion of nitrate to NH_3_. Zhou et al. developed a porous Ni foam-supported composite catalyst of Ru and NiOOH nanoparticles, achieving a FE close to 100% and excellent stability over 100 h [211].

Recent studies have further demonstrated that rational regulation of nitride-based catalysts through heterostructure construction and multimetallic coupling can effectively improve the activity and selectivity of eNO_3_^−^RR. For example, Dong et al. constructed a tandem Ag@Cu_3_N electrocatalyst by anchoring Ag nanoparticles on nitrogen-vacancy-rich Cu_3_N nanowires. Benefiting from the spatially separated tandem catalytic pathway, Ag sites preferentially catalyzed the initial deoxygenation of NO_3_^−^ to NO_2_^−^, whereas Cu_3_N sites promoted the subsequent hydrogenation of NO_2_^−^ to NH_3_. Consequently, the catalyst achieved an NH_3_ yield of 1.91 mmol h^−1^ cm^−2^ with an FE of 95.9% at −0.3 V vs. RHE. Furthermore, a Zn–NO_3_^−^ battery assembled with Ag@Cu_3_N delivered a maximum power density of 12.08 mW cm^−2^, demonstrating its potential for simultaneous ammonia synthesis and energy conversion [212]. Similarly, Kim et al. developed a bimetallic nickel molybdenum nitride (Ni_2_Mo_3_N/NF), which exhibited an FE of 97.72% and an NH_3_ yield of 5.55 mg h^−1^ cm^−2^. Mechanistic investigations revealed that the in situ formed amorphous Ni(OH)_2_ layer accelerated the conversion of NO_3_^−^ to NO_2_^−^, while the Mo–N species promoted the subsequent hydrogenation of NO_2_^−^ to NH_3_ [213]. These findings highlight that coupling transition-metal nitrides with surface reconstruction provides an effective strategy for optimizing the reaction pathway of eNO_3_^−^RR. In addition to metal nitrides, dynamic surface reconstruction of hydroxide and oxyhydroxide catalysts has recently attracted considerable attention. Lee et al. reported laser-decorated Pd/Co nanodendrites, which underwent in situ reconstruction into a Pd/CoOOH@Co heterostructure during NO_3_^−^RR. The reconstructed CoOOH@Co interface markedly enhanced the catalytic activity through synergistic interaction with Pd sites, enabling a maximum NH_3_ yield of 9.03 mg h^−1^ cm^−2^ and an FE of 92.35%. Moreover, a Zn–NO_3_^−^ battery based on the Pd/Co catalyst achieved an open-circuit voltage of 1.506 V and exhibited excellent durability over 500 h, demonstrating the practical potential of dynamically reconstructed hydroxide/oxyhydroxide electrocatalysts [214]. These recent studies demonstrate that integrating heterostructure engineering, multimetallic coupling, and dynamic surface reconstruction represents an effective strategy for improving the catalytic activity, selectivity, and long-term stability of metal nitride-, hydroxide-, and oxyhydroxide-based catalysts, providing new opportunities for practical electrocatalytic nitrate reduction.

### 3.7. Non-Metallic Catalysts

The widespread issue of metal ion leaching from metal-based catalysts and the associated environmental risks have driven the rapid development of non-metallic catalysts in the field of eNO_3_^−^RR to NH_3_. Ye et al. used laser-induced technology to prepare amorphous graphene catalysts, achieving an NH_3_ yield of 2859 μg h^−1^ cm^−2^ and an FE close to 100% at −0.93 V vs. RHE. DFT calculations indicated that the intrinsic defect structures of reconstructed graphene effectively promote electron transfer and optimize intermediate adsorption [215]. As a result, defect engineering strategies demonstrate significant advantages in the design of non-metallic catalysts. Additionally, a recent study showed that tuning the N vacancy concentration in carbon nitride via high-temperature calcination can significantly enhance eNO_3_^−^RR performance [216]. At the optimal N vacancy concentration, the FE and NH_3_ selectivity reached 89.96% and 69.78%, respectively, after 120 min of reaction in a 100 ppm NO_3_^−^ solution, with the mechanism linked to optimized adsorption energies of nitrogen-containing intermediates. Yu et al. prepared a biochar electrode using natural wood as a precursor, possessing a high HER overpotential, abundant O-containing groups, and sp^3^ C structure [217]. At −3.6 V vs. Hg/Hg_2_SO_4_, this electrode achieved a nitrate conversion rate of 91.2%, NH_3_ selectivity of 96%, and NH_3_ yield of 0.325 mmol h^−1^ cm^−2^. Yang et al. modified waste cigarette filters with polytetrafluoroethylene and pyrolyzed them to prepare fluorine-doped carbon materials (FC), achieving NH_3_ yield (23.8 mmol h^−1^ g^−1^) and FE (20%) that were 4 and 2 times higher than those of undoped carbon catalysts, respectively [218]. DFT simulations confirmed that F doping can effectively lower the energy barrier for NO_3_^−^ hydrogenation and suppress HER, providing theoretical guidance for the design of high-performance non-metallic electrocatalysts.

Developing efficient and stable non-metallic catalysts is a key direction for achieving the green and sustainable development of eNO_3_^−^RR technology. Future work should further elucidate the catalytic mechanisms of non-metallic active sites, optimize material structural stability, and promote the translation of laboratory findings to industrial applications.

## 4. Summary and Outlook

**Figure 12:**
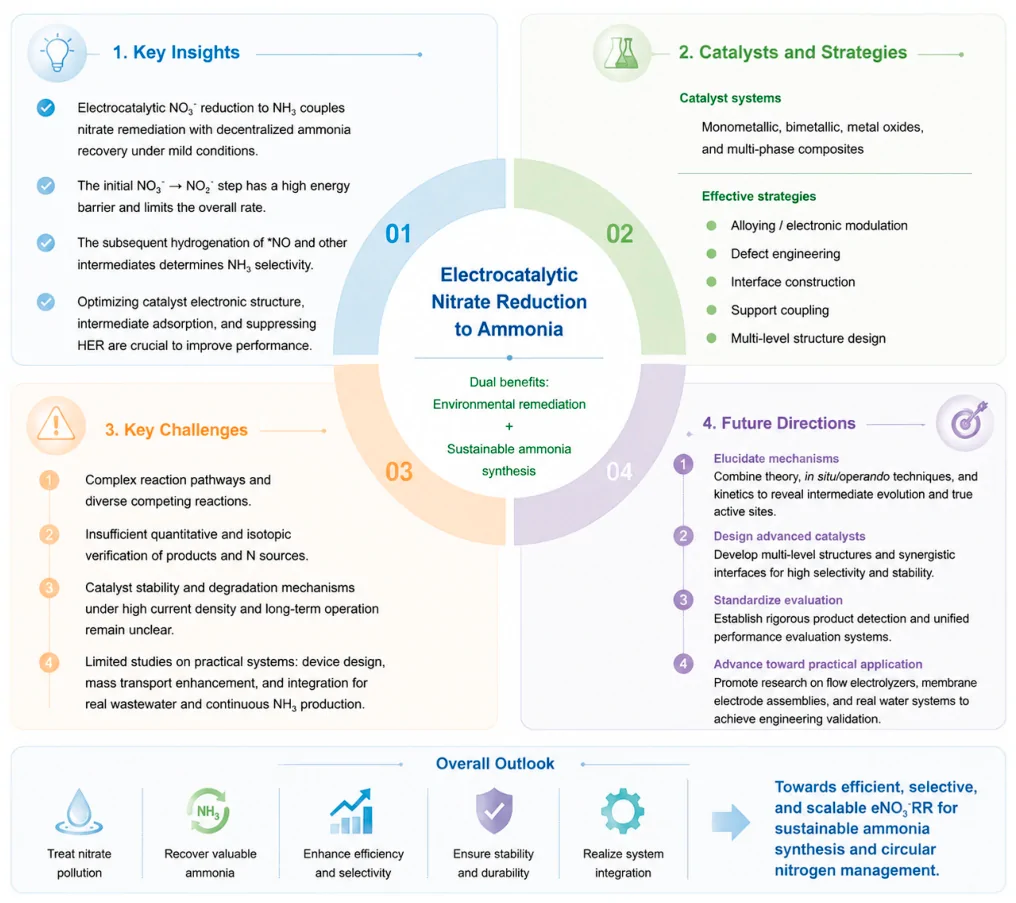
Summary and outlooks for the future advancement of electrocatalytic nitrate reduction to ammonia synthesis technology.

Figure 12 provides a detailed summary of the advancements in electrocatalytic nitrate reduction for ammonia synthesis technology, and offers insights into its future development directions. Details are as follows:

As a promising route for coupling nitrate remediation with decentralized ammonia recovery, the electrocatalytic nitrate reduction reaction enables the synergistic integration of nitrogen-containing pollutant treatment and ammonia resource recovery under mild conditions, thus being relevant to both environmental remediation and sustainable chemical production. In recent years, substantial progress has been made in understanding the reaction mechanism, designing catalytic materials, and tuning the performance of this reaction system. Existing research indicates that the initial conversion of nitrate to nitrite typically has a high energy barrier and is one of the key steps limiting the overall reaction rate. The subsequent protonation and hydrogenation behavior of adsorbed nitrogen-containing intermediates, especially *NO, plays a crucial role in determining the selectivity for ammonia generation. Therefore, rationally modulating the surface electronic structure of catalysts, optimizing the adsorption strength of key intermediates, and suppressing the competing hydrogen evolution reaction are central to enhancing the performance of eNO_3_^−^RR for ammonia production.

From the perspective of material systems, monometallic, bimetallic, metal oxide, and multi-phase composite catalysts have all shown promising research potential. Strategies such as alloying, defect engineering, interface construction, and support coupling can effectively improve the electronic environment of active sites, promote the directed conversion of reaction intermediates, and thus enhance ammonia yield and faradaic efficiency. However, several key challenges remain before this field can be applied practically. Firstly, the reaction pathway is complex, with many competing reactions; the distribution of products and the origin of nitrogen require more rigorous quantitative and isotopic verification. Secondly, the structural stability and performance degradation mechanisms of catalysts under high current densities and long-term operation are not yet well understood. Thirdly, current research is mostly focused on half-cell systems; studies on device configurations, mass transport enhancement, and system integration for practical nitrate-containing wastewater treatment and continuous ammonia production processes are still insufficient.

Future research should prioritize the following directions: (1) Combining theoretical calculations, in situ/operando characterization, and kinetic analysis to deeply elucidate the evolution pathways of key intermediates and the nature of true active sites; (2) developing novel catalytic materials with multi-level structures and synergistic interfaces to achieve high selectivity and high stability; (3) establishing more standardized product detection and evaluation systems to improve comparability and reliability among different research results; and (4) strengthening applied research in flow electrolyzers, membrane electrode assemblies, and real water systems to promote the transition of this technology from fundamental exploration to engineering validation. Overall, electrocatalytic nitrate reduction to ammonia holds dual significance for environmental remediation and green manufacturing. With a deepening understanding of catalytic mechanisms and continuous development of high-performance catalytic systems, the technology may find broader applications in sustainable ammonia synthesis.

## Figures and Tables

**Table 1:** Summary of the typical properties of representative transition-metal-based catalysts used in the eNO_3_^−^RR synthesis of NH_3_.

Catalyst	Electrolyte	NH_3_ Yield	Faradaic Efficiency (FE)	Selectivity	Ref.
Ru/POC	1 M Na_2_SO_4_ 0.2 mM NaNO_3_	1.336 mmol h^−1^ mg^−1^	96%	/	[43]
PdNDs/Zr-MOF	0.1 M Na_2_SO_4_ 500 ppm NO_3_^−^	287.31 mmol h^−1^ g^−1^	58.1%	/	[44]
BC_2_N/Pd	0.1 M KOH 250 mM KNO_3_	1730 μg h^−1^ cm^−2^	70.47%	/	[45]
Pd/NF	0.5 M Na_2_SO_4_ 0.1 M NaNO_3_	1.52 mmol h^−1^ cm^−2^	78%	/	[46]
nano-Ag	1 M KOH 14.3 mM NO_3_^−^	/	96.4%	89.6%	[47]
IrC-NCs	0.1 M HClO 0.2 M NaNO_3_	921 μmol h^−1^ mg^−1^	84.7%	/	[48]
Cu/PCNF	0.5 M Na_2_SO_4_ 0.05 M NaNO_3_	1.42 mol h^−1^ g^−1^	95.7%	/	[49]
Co NAs	1 M KOH 0.1 M KNO_3_	10.4 mmol h^−1^ cm^−1^	≥96%	/	[50]
Ni NPs	1 M NaOH 1 M NaNO_3_	15.49 mmol h^−1^ cm^−2^	93.0%	/	[51]
Ru_15_Co_85_ HND	0.1 M KOH 0.1 M KNO_3_	3.21 ± 0.17 mol g^−1^ h^−1^	97%	/	[52]
CuPd	0.4 M sodium phosphate buffer 100 mM NaNO_3_	258 μmol mg^−1^ h^−1^	>80%	/	[53]
Bi_1_Pd	1 M KOH 0.1 M NO_3_^−^	33.8 mg h^−1^ cm^−2^	≈100%	/	[54]
Cu_5_Fe_5_/OMC	0.1 M PBS 500 ppm KNO_3_	365.9 μg h^−1^ mg^−1^	73%	/	[55]
Cu_50_Ni_50_	1 M KOH 1700 ppm NO_3_^−^	/	~99.0%	/	[56]
Ni_1_Cu	0.5 M K_2_SO_4_ 200 ppm NO_3_^−^	326.7 μmol h^−1^cm^−2^	~100%	/	[57]
VCu-Au_1_Cu	0.1 M KOH 7.14 mM NO_3_^−^	555 μg h^−1^ cm^−2^	98.7%	/	[58]
Rh@Cu-0.6%	0.1 M Na_2_SO_4_ 0.1 M KNO_3_	21.61 mg h^−1^ cm^−2^	93%	/	[59]

**Table 2:** Summary of typical properties of representative metal oxides in the synthesis of NH_3_ from eNO_3_^−^RR.

Catalyst	Electrolyte	NH_3_ Yield	FE	Selectivity	Ref.
Cu/Cu_2_O	0.5 M Na_2_SO_4_	0.24 mmol h^−1^ cm^−2^	95.8%	81.2%	[179]
	200 ppm NO_3_^−^				
pCuO-10	0.05 M H_2_SO_4_	334 μmol h^−1^ cm^−2^	/	/	[115]
	0.05 M KNO_3_				
Pd-Cu_2_O CEO	0.5 M K_2_SO_4_	925.11 µg h^−1^ mg^−1^	96.56%	95.31%	[120]
	50 ppm KNO_3_				
PdCu/Cu_2_O	0.5 M Na_2_SO_4_	0.190 mmol h^−1^ cm^−2^	94.32%	96.70%	[121]
	100 ppm NO_3_^−^				
	0.1 M KNO_3_				
Cu_0.65_Pd_0.35_O_x_	0.25 M NO_3_^−^	1.41 mg h^−1^ cm^−2^	74%	/	[122]
Pd/TiO_2_	0.1 M PBS	1.12 mg h^−1^ cm^−2^	92.1%	/	[136]
Co@TiO_2_	0.1 M NO_3_^−^	800 μmol h^−1^ cm^−2^	96.7%	/	[138]
	200 ppm NO_3_^−^				
10Cu/TiO_2−x_	0.1 M Na_2_SO_4_	0.1143 mmol h^−1^ mg^−1^	81.34%	/	[139]
Co/CoO NSAs	200 ppm NO_3_^−^	194.46 μmol h^−1^ cm^−2^	93.8%	91.20%	[143]
	0.5 M K_2_SO_4_				
Co_3_O_4_-Mn_2_	0.1 M KNO_3_	35 mg h^−1^ cm^−2^	99.5%	/	[153]
	0.1 M Na_2_SO_4_				
CoMn_2_O_4_	0.1 M NO_3_^−^	1040.1 μg h^−1^ cm^−2^	92.4	/	[160]
	0.1 M K_2_SO_4_				
FeP/Fe_3_O_4_@C	0.1 mg-N L^−1^ KNO_3_	5.14 mg h^−1^ cm^−2^	98.1%	≈100%	[161]
	0.1 M KOH				
BiFeO_3_	0.1 M KNO_3_	90.45 mg h^−1^ mg^−1^	96.85%	/	[171]
	0.1 M Na_2_SO_4_				
Cu/MnO_x_	100 mM KNO_3_	5.53 mg h^−1^ mg^−1^	98.2%	/	[177]
	0.1 M NaNO_3_				
Fe_2_TiO_5_	Phosphate-buffered saline solution	0.73 mmol h^−1^ mg^−1^	87.6%	/	[178]

## Data Availability

No new data were created or analyzed in this study. Data sharing is not applicable to this article.
